# Flash Communication: Well-Defined Paramagnetic Polyhydrido-Bridged Iron–Iridium Clusters

**DOI:** 10.1021/acs.organomet.5c00301

**Published:** 2025-09-18

**Authors:** Zachary Dubrawski, Billie Shearman, Naïme Soulé, Erwann Jeanneau, Chloé Thieuleux, Clément Camp

**Affiliations:** † Laboratory of Catalysis, Polymerization, Processes and Materials (CP2M UMR 5128), CNRS, Universite Claude Bernard Lyon 1, CPE-Lyon, Institut de Chimie de Lyon, 43 Bd du 11 Novembre 1918, F-69616 Villeurbanne, France; ‡ Centre de Diffractométrie Henri Longchambon, Universite Claude Bernard Lyon 1, 5 Rue de la Doua, 69100 Villeurbanne, France

## Abstract

Polyhydrido transition metal complexes are a constant source of interest due to their variety in bonding and reactivity. Yet, well-defined paramagnetic polyhydrido systems are relatively rare given their instability. Herein, we extend the protonolysis reactivity of the Brønsted acidic Cp*IrH_4_ polyhydride toward transition metal silylamides. Specifically, we report the synthesis and characterization of a polyhydrido diiron tetrairidium paramagnetic cluster, [Fe­(Cp*IrH_3_)_2_]_2_, **1**, from the reaction between Fe­(HMDS)_2_(THF) and Cp*IrH_4_. Attempted reduction of this complex with potassium graphite yields instead an anionic species, K­[Fe­(Cp*IrH_3_)_3_], **2**, in a proposed disproportionation mechanism. Complex **2** can be independently synthesized in good yields upon treating **1** with K­[Cp*IrH_3_]. Attempts to synthesize Fe­(III) analogues from the oxidation of **1** or **2** failed, with oxidation occurring at the Ir centers. Likewise, reactions between Fe­(III) precursors and iridium polyhydride species consistently yield complex **1**. This highlights the unexpected driving force toward this very stable tetrairidium diiron paramagnetic polyhydride complex reported in this work.

## Graphical abstract

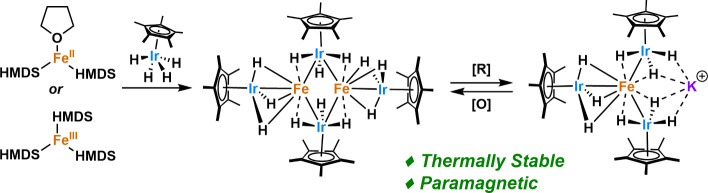

Polyhydrido transition metal clusters continue to fascinate inorganic chemists due to their diverse bonding patterns and interesting reactivity.^1–7^ The majority of polyhydrido compounds are diamagnetic. Paramagnetic counterparts remain relatively uncommon,^8–12^ mostly due to the relative instability of open-shell polyhydride species, which are prone to decomposition through a variety of mechanisms.^13–15^ Despite growing interest in iron catalysis, paramagnetic iron hydrido clusters are especially rare, with only a few reports in the literature (Table S1).^16–19^ The latter may yet offer new avenues for reactivity, as they are implicated in a number of enzymatic processes.^20–22^

In recent years, our research group has used the protonolysis reaction between pentamethylcyclopentadienyl tetrahydrido iridium­(V), Cp*IrH_4_, and a range of transition metal alkyl complexes, to prepare heterobimetallic polyhydride species.^23–27^ Herein we expand this strategy to silylamido derivatives,^28^ and we report the synthesis, characterization, and reactivity of paramagnetic polyhydrido bridged iron–iridium clusters.

Addition of 2 equiv of Cp*IrH_4_ to bis­((trimethylsilyl)­amido)­iron­(II) tetrahydrofuran adduct, Fe­(HMDS)_2_(THF), in pentane at room temperature generates complex [Fe­(Cp*IrH_3_)_2_]_2_, **1**, isolated as a bright yellow-orange solid in 85% yield (Scheme 1). The ^1^H NMR spectrum of complex **1** shows two paramagnetically shifted signals at 26.2 and 17.7 ppm (C_6_D_6_, 298 K, 300 MHz) appearing as two 1:∼1 broad singlets, and presumably corresponding to two Cp* ligands in different chemical environments (Figure S1). The hydride ^1^H NMR resonances are not observed in a range of +4000 to −4000 ppm, as is often the case for hydride ligands located at close proximity of paramagnetic centers.^29–32^

**Scheme 1:**
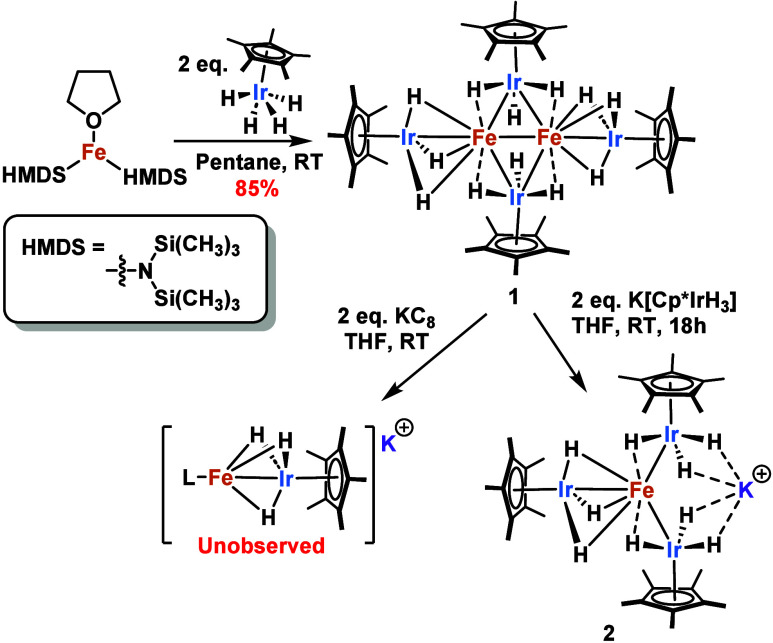
Synthesis of [Fe_2_Ir_4_] and [FeIr_3_] Polyhydrido Clusters^a^

a The proposed hydride bridging modes are inferred from the solid-state structures (see below).

The diffuse reflectance infrared Fourier transform (DRIFT) spectrum for **1** (Figure S15) reveals ν­(M–H) signals at 2244–1789 cm^–1^ consistent with the presence of metal hydrides in various coordination environments (see below). Complex **1** is electron paramagnetic resonance (EPR) spectroscopy silent when performed at the X band either at room temperature or at 77 K. Solution-state Evans method measurement in C_6_D_6_ yields a magnetic moment of 4.5 μ_B_ (Figure S2). Iron clusters are known for their significant ferro- or antiferromagnetic coupling properties, and so we cannot directly correlate this value to a specific spin-state configuration.^33–35^

Single crystal X-ray diffraction analysis revealed a tetrairidium diiron cluster structure, shown in Figure 1. The dimeric nature is not surprising given the propensity of [Cp*IrH_*x*_]^*y*−^ moieties to bridge several metal centers, as well as the tendency of nonsolvato Fe­(HMDS)_2_ systems to dimerize.^23,24,36,37^ However, the majority of these di-iron, diamond core-like structures show the two Fe atoms and other coordinating atoms (N, S, O or other metals) from the bridging ligands lying along the same plane of the molecules.^16,37–40^ Here, we see a significant deflection from this plane evidenced by the Ir2–Fe1–Fe2–Ir3 dihedral angle of 156.56°, which we propose is due to a terminal hydride deflecting the Fe center away from the butterfly cluster core (see Figure S18 for a better view of the saddle-shaped core). Unfortunately, hydrides located adjacent to very heavy elements such as Ir cannot be accurately determined from the Fourier difference map and as such have not been represented here.^41^ However, from the solid-state structure we can infer their locations from the Cp*–Ir–Fe angles, as has been previously reported.^42,43^ The Cp*_centroid_–Ir1–Fe1 angle of 173° is consistent with structures having three bridging hydrides between the two transition metals whereas the Cp*_centroid_–Ir2–Fe_1,2 centroid_ angle of 163° tentatively confirms different bridging modes within the architecture, perhaps indicative of a terminal hydride. The Ir–Fe bond lengths are quite long (2.541(2)–2.671(2) Å) but comparable to other Fe–Ir pairs with bridging hydrido ligands.^44,45^ The Formal Shortness Ratio (FSR, defined as the ratio of interatom distance to the sum of atom radii) can be used as a qualitative guide for intermetallic bonding. With an FSR over unity (FSR = 1.04), we conclude that **1** does not exhibit any Fe–Ir bonding. Similarly, the very long Fe1–Fe2 distance (2.898(3) Å, FSR = 1.24) precludes intermetallic bonding and suggests no hydride bridges between the iron centers.^17,18^ Incidentally, other Fe:Ir stoichiometries (Fe_2_(Cp*IrH_3_)­(HMDS)_3_ and Fe_2_(Cp*IrH_3_)_2_(HMDS)_2_) could be obtained (crystal structures showing atom connectivity are located in Figures S19 and S20) which also show these dihedral deflections (suggestive that it is the bridging Cp*IrH_3_^–^ moieties causing this effect).^46^ However, these partially substituted systems are not fully characterized and are not explored further in this report.

**Figure 1:**
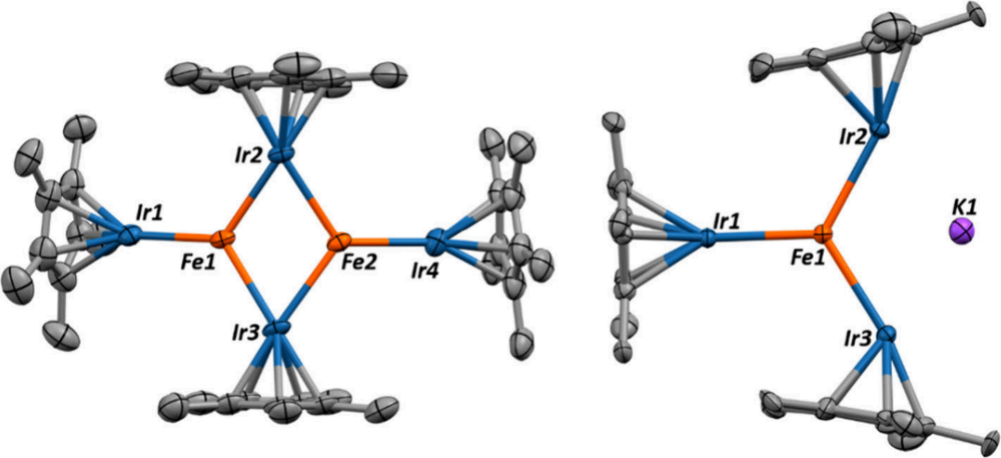
X-ray crystal structures of complexes **1** (left) and **2** (right). Thermal ellipsoids are shown at 50% probability levels, and hydrogen atoms are removed for clarity. Relevant bond lengths and angles for complex **1**: Fe1–Ir1 = 2.541(2) Å (FSR = 1.04), Fe1–Ir2 = 2.671(2) Å (FSR = 1.10), Fe1–Fe2 = 2.898(3) Å (FSR = 1.24), Cp*_centroid_–Ir1–Fe1 = 173.4(1)°, Cp*_centroid_–Ir2–Fe_1,2 bond centroid_ = 163.3(1)°, Ir1–Fe1–Fe2 = 158.3(1)°, Ir2–Fe1–Fe2–Ir3 = 154.6(1)°, τ_4, Fe1_ = 0.53. Relevant bond lengths and angles for complex **2**: Ir1–Fe1 = 2.575(2) (FSR = 1.06), Ir2–Fe1 = 2.665(2) (FSR = 1.09), Ir3–Fe1 = 2.665(2) (FSR = 1.09), Ir1–K1 = 3.628(2), Ir3–K1 = 3.741(2), < Cp*_centroid_–Ir1–Fe1 = 137.3(1)°, < Cp*_centroid_–Ir2–Fe1 = 178.7(1)°, < Cp*_centroid_–Ir3–Fe1 = 137.3(1)°. Ir2–Fe1–K1 = 144.1(1)°.

Complex **1** is remarkably stable. No detectable degradation was observed after months in an ambient glovebox atmosphere or in a benzene solution, even after heating to 70 °C for multiple days. Unfortunately, **1** is also stable toward substrates: no reaction is observed with simple small molecules such as alkenes, alkynes, azides, isocyanates, N_2_, CO_2_, or CO under thermal or photochemical conditions. With the interest of exploring other oxidation states of iron within this architecture, we turned to reduction with 2 equiv of potassium graphite (KC_8_). The reaction can be monitored by ^1^H NMR spectroscopy in THF-*d*_8_ yielding a new compound, **2**, featuring one large signal at +33 ppm. A single crystal of product **2** could be obtained from a saturated pentane:THF solution at −40 °C, which reveals the formation of an anionic iron tris-iridate complex with a potassium countercation, K­[Fe­(Cp*IrH_3_)_3_] (Scheme 1). The mass balance of the material isolated (24% yield) at the end of this reaction revealed a significant loss. We therefore assume some disproportionation mechanism is at play; i.e. the elusive Fe^I^ iridate complex falls apart into an Fe^0^ fragment and **2**. Performing this reduction under an atmosphere of dinitrogen instead of argon, in the hope of an N_2_ adduct, unfortunately does not alter the outcome.

The crystal structure of **2** (Figure 1) reveals an infinite chain of potassium atoms ligated by the FeIr_3_ unit. The Fe–Ir bond distances of 2.662–2.579 Å (FSR = 1.09–1.06) have not substantially changed from complex **1**. The most interesting structural difference is the dramatic change in the Cp*_centroid_–Ir1–Fe1 angle, which has bent from 163° in complex **1** to 135° in **2**. This rotation of the Cp* ligands suggests the hydrides extend out from the Ir center, in the direction of the potassium center, most likely as a result of hydride–cation bonding.^17,47,48^ Given the single signal in the ^1^H NMR data (which we attribute to the Cp* ligands), we presume some fluxionality of this structure in solution, as already seen in other asymmetric (Cp*IrH_3_)^−^ derived polynuclear species.^25,42,43^

**2** can be independently synthesized through the addition of 2 equiv of K­[Cp*IrH_3_] to **1** in THF in 78% yield.^25^ The paramagnetic ^1^H NMR signal (+33 ppm) for the isolated complex matches that observed during ^1^H NMR reaction monitoring of the KC_8_ reduction of **1**, and single crystals (grown from a saturated THF:pentane mixture) match the unit cell obtained previously. Solution state Evans’ method calculations reveal a magnetic moment of 4.8 μ_B_ corresponding to four unpaired electrons, which we assign as a high-spin Fe­(II) center. Like **1**, complex **2** is EPR silent. Despite the ability of K­[Cp*IrH_3_] to break the dimeric structure of **1**, the strongly coordinating N-heterocyclic carbene 1,3-bis­(2,6-diisopropylphenyl)-2-imidazolidinylidene (IPr) does not react with **1** to give a monomeric species.

Given the complex reduction reactivity of **1**, we turned to oxidation (Scheme 2). The reaction of **1** with AgPF_6_ in THF proceeds readily to yield benzene insoluble paramagnetic species with two signals at 18.3 and 14.8 ppm in the ^1^H NMR spectrum collected in THF-*d*_8_ (Figure S8). After several attempts with different crystal growing conditions, one crystal suitable for single crystal diffraction was obtained and revealed the formation of a cationic Ag­(I) adduct with complex **1** rather than an oxidation product (Figure S22). We conclude that the bulk material contains a mixture of indistinct Fe, Ir and Ag coordination polymers, and did not pursue further. Oxidation of complex **2** with 1 equivalent of AgPF_6_ leads to the formation of **1** and the sparingly soluble, polymeric [Ag­(Cp*IrH_3_)]_*n*_ complex previously reported by our group,^42^ presumably as a result of transmetalation from K^+^ to Ag^+^. The remaining (Cp*IrH_3_)_2_Fe fragment dimerizes in solution to yield complex **1**, as confirmed by ^1^H NMR reaction monitoring (see the Supporting Information (SI)). These results suggest that silver salts are not oxidizing enough, prompting us to explore stronger oxidizing agents.

**Scheme 2:**
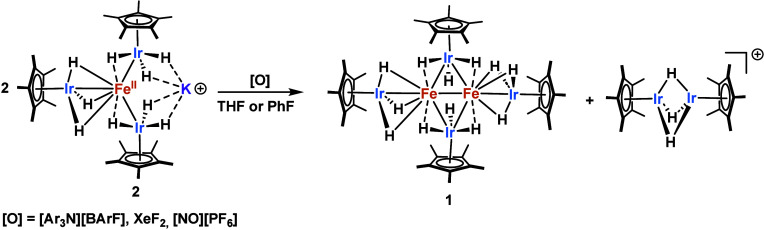
Oxidation of Complex **2**

The reaction of tris­(4-bromophenyl)­ammoniumyl tetrakis­(pentafluorophenyl)­borate ([Ar_3_N]­[BArF], Magic Blue, MB) with **2** leads not only to the consumption of the organic radical cation but also to the production of complex **1**. Here we presume that MB oxidizes one [Cp*IrH_3_]^−^ fragment that dissociates from the Fe center. Crystals grown from this mixture confirm this hypothesis through the observation of a cationic hydrido-bridged Ir dimer, [Cp*Ir­(μ-H)_3_IrCp*]­BArF (Figure S23).^49^ Analogous to the Ag case above, the liberated (Cp*IrH_3_)_2_Fe fragment dimerizes in solution to produce **1** without a formal Fe oxidation state change. Addition of XeF_2_ and [NO]­[PF_6_] produce similar results (Figure S11). Unfortunately compound **2** is not stable in more coordinating solvents such as acetonitrile, most likely due to the dissociation of K­[Cp*IrH_3_] and formation of **1** (Figure S12), limiting choice of reaction conditions.

The inability for the hydrido-bridged Fe­(II) clusters **1** and **2** to be oxidized led us to attempt the direct synthesis of Fe­(III) congeners via salt metathesis between FeCl_3_ and 3 equiv of K­[Cp*IrH_3_]. Perhaps unsurprisingly, complex **1** emerged as a major product of this reaction. We assume here that 1 equiv of K­[Cp*IrH_3_] is acting as a reductant. Similarly, the reaction of Fe­(HMDS)_3_ with 3 equiv of Cp*IrH_4_ proved unselective, yet again yielding complex **1** as a major product, as confirmed by ^1^H NMR. It is clear from these results that the formation of the very stable diiron cluster, **1**, is the driving force in all this chemistry, preventing further reactivity.

Overall, this work explores structural variations in heterometallic Ir/Fe complexes, expanding the currently limited examples of iron-containing heterometallic polyhydride clusters through a protonolysis reaction of Cp*IrH_4_ with an iron­(II) silylamide. The [Cp*IrH_*x*_] fragment, whose protonolysis reactivity was previously limited to reaction with metal alkyls, acts as a versatile weak-field ligand adopting what we propose to be one, two, or three bridging hydrido bonding modes. This flexibility can yield compounds of various nuclearities, highlighting the diversity of this heterobimetallic synthesis strategy. Given the commercial availability of transition metal (silyl)­amides across the periodic table, we anticipate rapid development in hydrido-bridged heterobimetallic chemistry.

## Supplementary Material



## Notes

General considerations, mono- and multimetallic iron polyhydrido complexes, synthetic procedures, and NMR, DRIFT, XRD, and ESI-MS data (PDF)
