# Supplementary material for: Flash Communication: Well-Defined Paramagnetic Polyhydrido-Bridged Iron–Iridium Clusters
Source: Organometallics. 2025 Sep 18;44(19):2151–5. doi: 10.1021/acs.organomet.5c00301 (PMC12522137; doi:10.1021/acs.organomet.5c00301)
Supplement: Supplementary file 1 [file om5c00301_si_001.pdf]

Supporting Information

Flash Communication: Well-Defined Paramagnetic Polyhydrido-Bridged Iron-Iridium Clusters

Zachary Dubrawski,[a] Billie Shearman,[a] Naime Soulé,[a] Erwann Jeanneau,[b] Chloé Thieuleux,[a] Clément Camp[a,]*

[a] Laboratory of Catalysis, Polymerization, Processes and Materials (CP2M UMR 5128), CNRS, Universite Claude Bernard Lyon 1, CPE-Lyon, Institut de Chimie de Lyon, 43 Bd du 11 Novembre 1918, F-69616 Villeurbanne, France. E-mail: clement.camp@univ-lyon1.fr

[b] Centre de Diffractométrie Henri Longchambon, Universite Claude Bernard Lyon 1, 5 Rue de la Doua, 69100 Villeurbanne, France

Table of Contents:

General Experimental Considerations 3

Syntheses 4

Tris(bis(trimethylsilyl)amide) Iron(III) 4

Synthesis of $[Fe(Cp^*IrH_3)_2]_2$, **1** 4

Reduction of **1** with 2 equivalents of KC_8 5

Reaction of **1** with $K[Cp^*IrH_3]$ to generate complex $K[Fe(Cp^*IrH_3)_3]$, **2** 5

Attempted oxidation of **1** with $AgPF_6$ 6

General procedure for the attempted oxidations of **2** 6

Attempted synthesis of Fe(III) analogues *via* salt methathesis using $FeCl_3$ and $K[Cp^*IrH_3]$ 7

Attempted synthesis of Fe(III) analogues *via* protonolysis using $Fe(HMDS)_3$ and Cp^*IrH_4 7

NMR Spectra 8

Diffuse Reflectance Infrared Transmission (DRIFT) Spectra 16

Supplementary X-Ray Crystal Structures 18

X-Ray Crystallography Tables 21

Supplementary ESI-MS data 23

References 25

Table S 1 : Recent examples of monometallic and multimetallic iron polyhydrido species			
Entry	Complex	Spin State	Reference
1	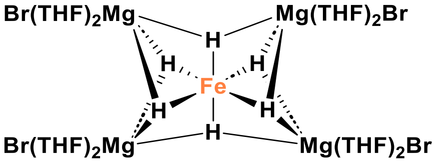	unknown	1
2	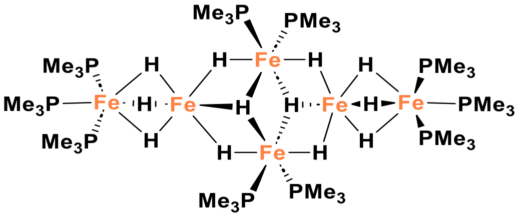	Paramagnetic	2
3	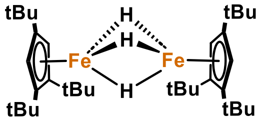	Paramagnetic	3
4	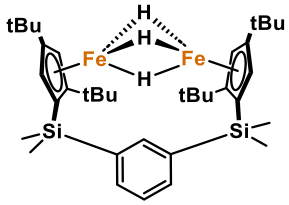	Paramagnetic	4
5	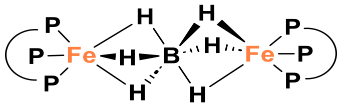	Diamagnetic	5
6	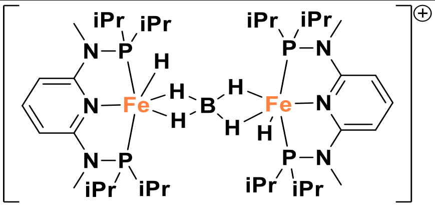	Diamagnetic	6
7	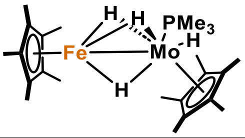	Diamagnetic	7
8	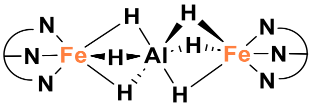	Diamagnetic	8
9	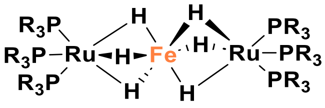 R = pTol	Paramagnetic	9
10	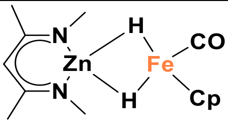	Diamagnetic	10
11	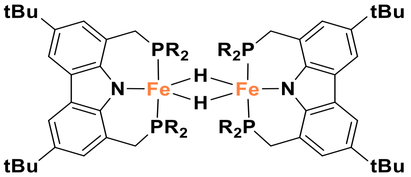	Paramagnetic	11

General Experimental Considerations

Unless otherwise noted, all reactions were performed either using standard Schlenk line techniques or in an MBRAUN glovebox under an atmosphere of purified argon (<1 ppm of O_2/H_2O). Glassware and cannulas were stored in an oven at ~100 °C for at least 16 h prior to use. THF and *n*-pentane were purified by passage through a column of activated alumina, dried over Na/benzophenone, vacuum-transferred to a storage flask, and freeze–pump–thaw degassed prior to use. Deuterated solvents (toluene-d_8, THF-d_8, and C_6D_6) were dried over Na/benzophenone, vacuum-transferred to a storage flask, and freeze–pump–thaw degassed prior to use. Pyridine-d_5 was dried over calcium hydride, vacuum-transferred to a storage flask, and freeze–pump–thaw degassed prior to use. The syntheses of Cp*IrH_4 and $Fe(HMDS)_2(THF)$ were carried out following literature procedures.[12,13] The synthesis of K[Cp*IrH_3] was performed following a literature procedure.[14] The synthesis of $Fe(HMDS)_3$ follows a modified literature procedure and is discussed below.[15] The synthesis of Magic Blue with a tetrakis(pentafluorophenyl)borate anion was performed following a literature procedure.[16] All other reagents were acquired from commercial sources and used as received.

IR Spectroscopy

The samples were prepared in a glovebox (either pure crystalline material, or diluted in dry KBr powder), sealed under argon in a Diffuse Reflectance Infrared Fourier Transform (DRIFT) cell fitted with KBr windows, and then analyzed using a Nicolet 670 FT-IR spectrometer.

Elemental Analyses

Elemental analyses were performed under an inert atmosphere at Mikroanalytisches Labor Pascher, Germany.

X-Ray Diffraction - Structural Determinations

Suitable crystals were coated in parabar oil, selected manually under a binocular microscope and mounted on a Rigaku-OD Synergy-S single-crystal diffractometer equipped with an Hypix-100 detector. Intensities were collected at 100K with molybdenum radiation (λ=0.71073 Å) for all crystals examined in this work.[17] Reflection indexing, unit-cell parameters refinement, Lorentz-polarization correction, peak integration and background determination were carried out with the CrysalisPro software.[17] An analytical absorption correction was applied using the modeled faces of the crystal.[18] The resulting set of *hkl* was used for structure solution and refinement. The structures were solved with the ShelXT structure solution program using the intrinsic phasing solution method and by using Olex2 as the graphical interface.[19,20] The model was refined with version 2018/3 of ShelXL using least-squares minimization.[19]

CCDC 2442205-2442209 contain the supplementary crystallographic data for this paper. These data can be obtained free of charge from The Cambridge Crystallographic Date Centre via www.ccdc.cam.ac.uk/data_request/cif.

NMR Spectroscopy

Solution NMR spectra were recorded on a Bruker AV-300 spectrometer. ^{1}H and ^{13}C chemical shifts were measured relative to residual solvent peaks, which were assigned relative to an external TMS standard set at 0.00 ppm. ^{1}H and ^{13}C NMR assignments were confirmed by ^{1}H–^{1}H COSY, ^{1}H–^{13}C HSQC, and HMBC experiments. NMR data recorded as follows: chemical shift (δ) [multiplicity, coupling constant(s) *J* (Hz), relative integral], where multiplicity is defined: s = singlet, d = doublet, t = triplet, q = quartet, m = multiplet or combinations thereof, and prefixed br = broad. Evans method calculations were performed using diamagnetic corrections from the literature.[21]

Syntheses

Tris(bis(trimethylsilyl)amide) Iron(III)

This synthesis is adapted from literature.[15]

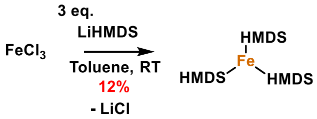

In an argon filled glovebox, a 150 mL Schlenk flask was charged with 6.23 g of $FeCl_3$ (38 mmol, 1 eq.) which was suspended in 75 mL toluene. A second 500 mL Schlenk flask was charged with 18.99 g of LiHMDS (113 mmol, 3 eq.) and 100 mL of toluene were added while stirring to fully solubilize the salt. Both flasks were connected to a Schlenk line and both solutions were cooled to 0°C with the use of an ice bath. The $FeCl_3$ solution was transferred dropwise to the LiHMDS solution *via* the use of a stainless steel cannula. The resulting black solution was allowed to stir at room temperature for 7 days under an argon atmosphere. The resulting dark green suspension was heated to reflux for 1 hour before filtration *via* the use of a Schlenk fritted glass filter to remove the precipitated LiCl. The volatiles were removed under reduced pressure at room temperature and the oily green material was brought back into the glovebox. The solid was washed with cold (ca -40°C) toluene to yield a sticky green solid, which was then sublimed at 10^{-5} mbar with the use of a heatgun, using a sublimating apparatus with a cold finger connected to a high-vacuum line. The collected solid was triturated with cold (ca -40°C) pentane and dried under reduced pressure to yield 2.44 g of a sticky pale green material (12% yield). This purification procedure was found to be necessary to avoid any ^{7}Li NMR signals arising from LiHMDS or LiCl impurities.

^{1}H NMR (300 MHz, C_6D_6) δ -3.27 ppm (br s)

Synthesis of $[Fe(Cp^*IrH_3)_2]_2$, **1**

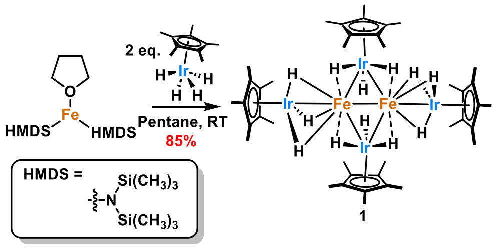

In an argon filled glovebox, a 20 mL scintillation vial was charged with 0.050 g of Cp^*IrH_4 (0.15 mmol, 2 eq) and dissolved in 15 mL pentane. 0.034 g $Fe(HMDS)_2(THF)$ (0.075 mmol, 1 eq) was added portion wise with vigorous stirring resulting in an immediate colour change to a deep orange. The solution was left to react overnight at room temperature before the volatile materials were removed under reduced pressure. The orange material was triturated with a small volume (10 mL) of cold (ca -40°C) pentane to yield analytically pure **1** (95% yield). Single crystals (orange blocks) for X-ray diffraction were grown from a saturated pentane solution cooled to -40 °C.

^{1}H NMR (300 MHz, C_6D_6) δ 26.3 (s, 30H, CH_3 (Cp*)), 17.7 (s, 30H, CH_3 (Cp*))

Evans Method (C_6D_6, 300 MHz): 4.5 μ_B

DRIFT: 3025 – 2903 (C-H), 2244 – 1789 (M-H) cm^{-1}

Elemental Analysis: Calculated for $Fe_2Ir_4C_{40}H_{60}$: C 33.56, H 4.93. Experimentally: 33.32 H 4.92

Reduction of **1** with 2 equivalents of KC_8

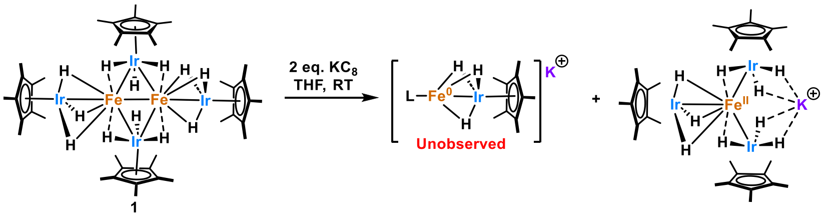

In an argon filled glovebox a 20 mL scintillation vial was charged with **1** (35 mg, 2.44x10^{-5} mol, 1 eq.) and dissolved in 5 mL THF. To this rapidly stirring solution was added 2 eq. of KC_8 in one portion (7 mg, 5.92 x10^{-5} mol, 2.2 eq.) at room temperature and the solution was left to stir for 3h before filtration through a glass fiber filter. The solvent was removed under reduced pressure yielding a yellowish solid, readily soluble in THF and ethers but insoluble in pentane. The solid was triturated with pentane, collected on a filter and washed with more pentane. Dissolving the solid through washing the filter with THF and removal of the solvent under reduced pressure yielded 6.3 mg of complex **2** (24% yield). X-ray quality single crystals were grown from vapour diffusion of pentane into a saturated THF solution at -40 °C.

^{1}H NMR (300 MHz, THF-d_8) δ 33.3 (s, 45H, CH_3 (Cp*))

Reaction of **1** with K[Cp*IrH_3] to generate complex K[Fe(Cp*IrH_3)$_3$], **2**

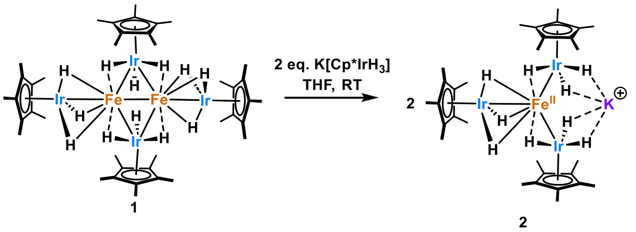

Cp*IrH_4 (26.2 mg, 0.079 mmol, 1.0 eq.) and benzyl potassium (11.0 mg, 0.084 mmol, 1.05 eq.) were added to a 20 mL scintillation vial with THF (2 mL) to generate a solution of K[IrCp*H_3]. Separately, **1** (56.7 mg, 0.040 mmol, 0.5 eq.) was dissolved in THF (2 mL) forming a dark orange solution and added dropwise to the colourless K[IrCp*H_3] solution with immediate colour change from dark orange to yellow. After stirring at room temperature for 12 h, the volatiles were removed under reduced pressure. The crude yellow solid was triturated with *n*-pentane (10 mL), dissolved in a minimal volume of THF and cooled to -40 °C overnight, affording thin yellow plates (28.7 mg, 33% yield). Repeating this recrystallization with the mother liquor obtained allows for the isolation of more **2** with a total yield of 67.1 mg after three crystallizations. Yield = 78% .

^{1}H NMR (300 MHz, THF-d_8) δ 33.3 (s, 45H, CH_3 (Cp*))

Evans Method (THF-d_8, 300 MHz): 4.8 μ_B

DRIFT: 2974 - 2903 (C-H), 2005 - 1893 (M-H)

EA: Calculated for $C_{30}H_{54}FeIr_3K$: C 33.17, H 5.01. Found: C 32.60, H 4.80

Attempted oxidation of **1** with $AgPF_6$

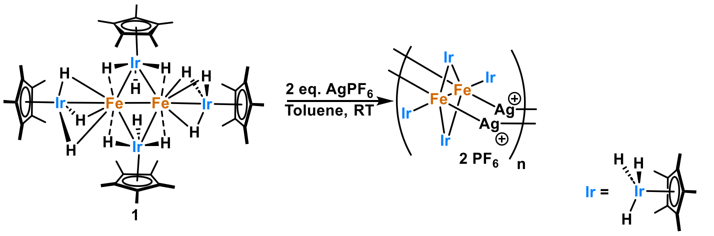

In an argon filled glovebox, a 20 mL scintillation vial was charged with 0.051 g of complex **1** ($3.56x10^{-5}$ mol, 1 eq.) and dissolved in 10 mL toluene. To this solution was added 0.018 g ($7.12x10^{-5}$ mol, 2 eq.) $AgPF_6$ in one portion. The solution was left to stir overnight at room temperature to yield a dark brown suspension in a pale yellow supernatant. The dark brown solid was filtered, washed with pentane and dried under reduced pressure to yield 0.062 g of a crude material. Large crystals could be grown from the slow diffusion of pentane into a saturated THF solution. Despite the crystalline appearance, these crystals could not be used for XRD as crystals thought to be single either did not diffract on the instrument or a unit cell could not be found despite strong diffraction spots. After several attempts with several crystal growing conditions, one crystal was found to diffract (grown from a pentane vapour diffusion into a saturated fluorobenzene solution) to yield the structure given below. This structure is likely not representative of the whole bulk material, which we suspect is a polymeric metal coordination polymer. Nevertheless, it reveals that silver cations lack sufficient oxidizing power and instead coordinate to the hydride structure. Given the ill-defined structure of the material, we have not carried out Evans method NMR characterization.

^{1}H NMR (300 MHz, THF-d8) δ 18.3 (br s, 30H), 14.7 (br s, 30H)

DRIFT: 3024 - 2778 (C-H), 2041 - 1667 (M-H)

General procedure for the attempted oxidations of **2**

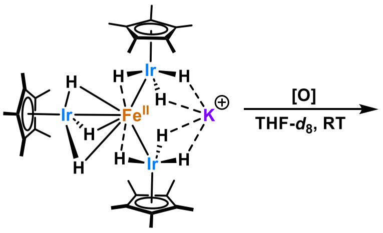

2

[O] = $AgPF_6$, XeF_2, $[Ar_3N][BArF]$ = Magic Blue, $[NO][PF_6]$

In an argon filled glovebox, a J-Young teflon capped NMR tube was charged with 0.010 - 0.015 g of complex **2** and dissolved in ~0.5 mL THF-*d*$_8$. To this tube was added one equivalent of the chosen oxidant and the reaction monitored by ^{1}H NMR spectroscopy. In all cases, complex **1** was observed from the reaction, usually with the co-production of Cp^*IrH_4 as observed by ^{1}H NMR. In the case of $AgPF_6$, the previously reported polymeric $[Cp^*IrH_3Ag]_n$ was observed precipitating from the reaction mixture.[22] This observation was confirmed by first removing all volatile materials from the NMR tube under reduced pressure, then redissolving the reaction mixture in pyridine-*d*$_5$. ^{1}H NMR (400 MHz, pyridine-*d*$_5$, 293 K) δ 3.6 (s, 15H, CH_3 (Cp*)), −14.2 (s, 3H, H–Ir).

Attempted synthesis of Fe(III) analogues *via* salt methathesis using $FeCl_3$ and K[Cp*IrH_3]

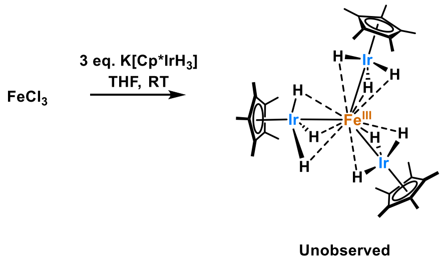

Cp*IrH_4 (28.9 mg, 0.087 mmol, 1.0 eq.) and benzyl potassium (12 mg, 0.092 mmol, 1.05 eq.) were added to a 20 mL scintillation vial with THF (2 mL) to generate a solution of K[IrCp*H_3]. This solution was added dropwise to a rapidly stirring pale green solution of 5.0 mg (0.031 mmol, 0.35 eq) anhydrous $FeCl_3$ in 2 mL THF. The dark brown/orange solution was left to stir for 3h at room temperature before the volatiles were removed under reduced pressure. ^{1}H NMR analysis of the crude solid in THF-d_8 showed typical signals for complexes **1**, **2** and unreacted Cp*IrH_4.

Attempted synthesis of Fe(III) analogues *via* protonolysis using $Fe(HMDS)_3$ and Cp*IrH_4

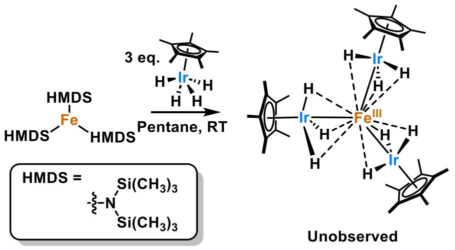

In an argon filled glovebox, a 20 mL scintillation vial was charged with 0.025 g $Fe(HMDS)_3$ ($4.7x10^{-5}$ mol, 1 eq.) and dissolved in 3 mL pentane. To this rapidly stirring solution was added dropwise a solution of 0.0461 g Cp*IrH_4 ($1.4x10^{-4}$ mol, 3 eq.) in 3 mL pentane with a rapid colour change to dark orange-brown. The solution was left to stir at room temperature overnight before the volatiles were removed under reduced pressure to yield 0.032 g of a dark brown/orange powder. ^{1}H NMR analysis of the crude solid showed typical signals for complexes **1**, **2** and unreacted Cp*IrH_4.

NMR Spectra

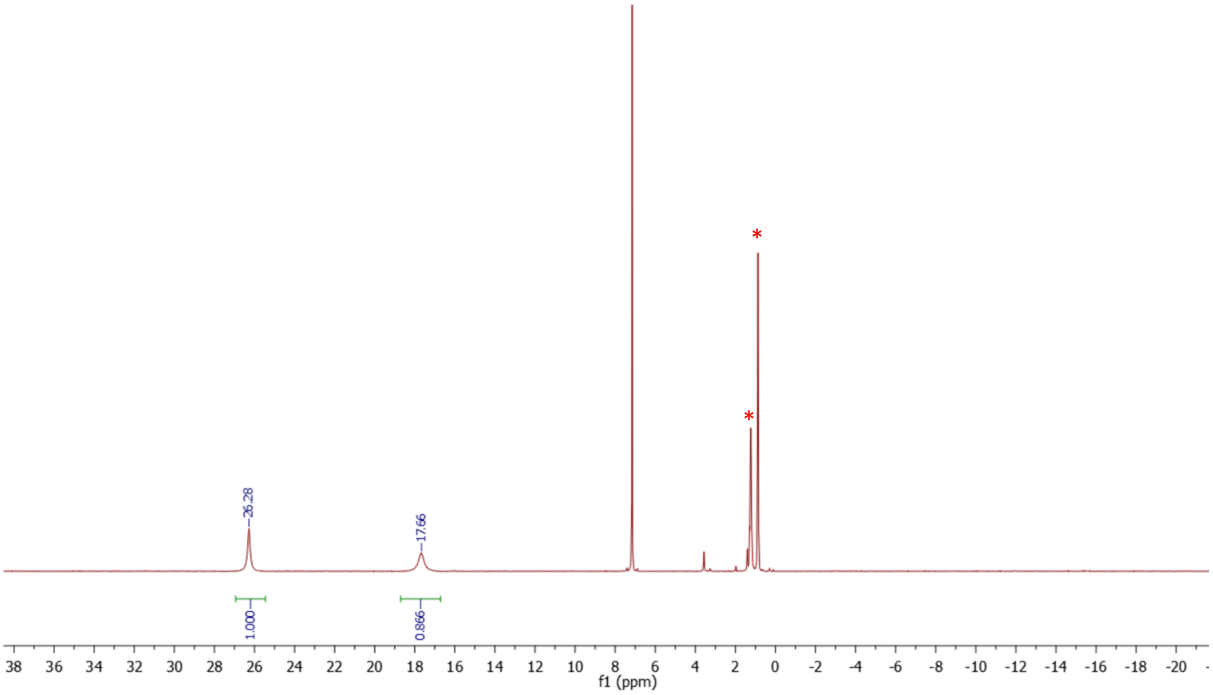

Figure S 1 : ^{1}H NMR spectrum of compound 1 in C_6D_6 solution (298K, 300 MHz). Some pentane and THF are still visible in the spectrum and are marked with an asterisk (*).

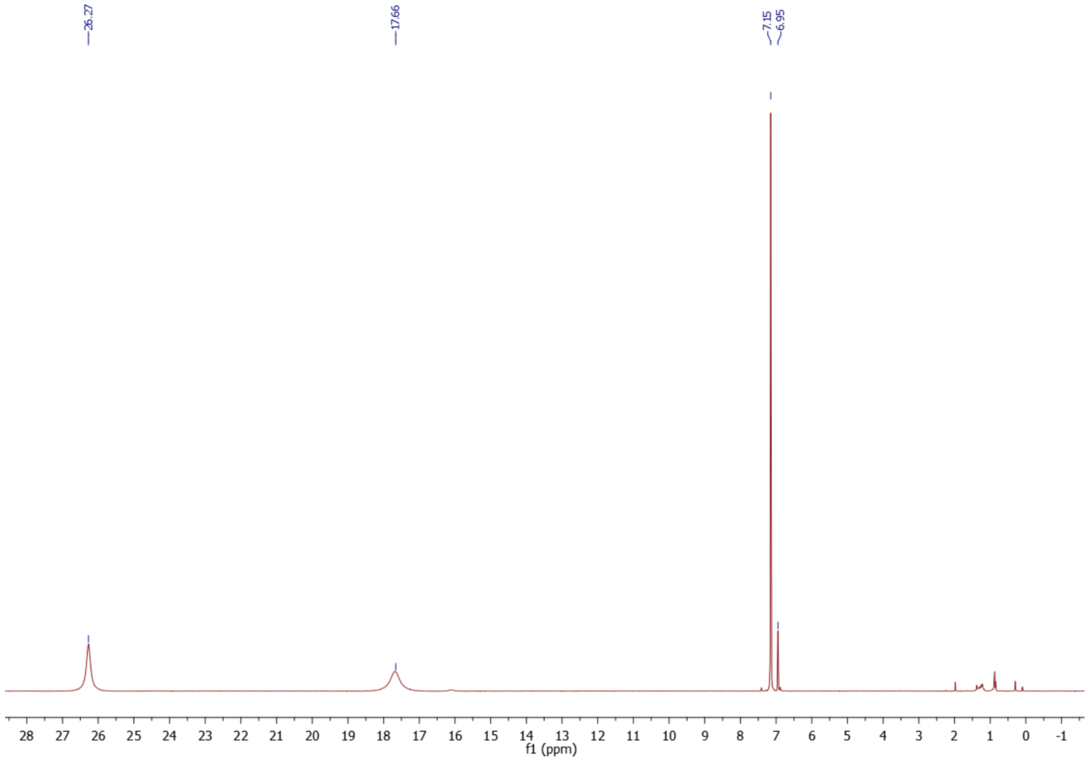

Figure S 2: ^{1}H NMR spectrum of complex 1 recorded in C_6D_6 solution (300 MHz, 298 K, 6.0 mM) with a capillary C_6D_6 insert for Evans Method calculation of the magnetic moment. The C_6D_6 signal has been shifted by 0.20 ppm (61 Hz) corresponding to a magnetic moment of 4.5 μ_B.

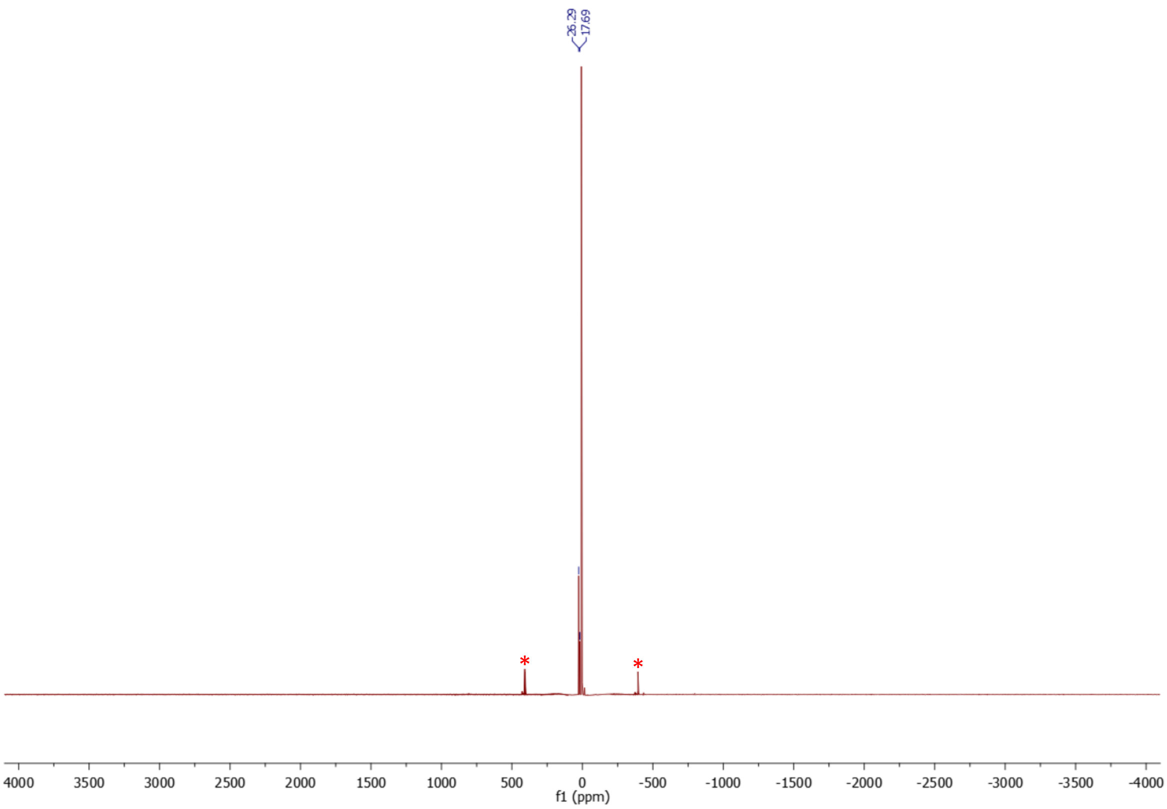

Figure S 3 : The ^{1}H NMR spectrum of complex 1 in C_6D_6 (300 MHz, 298 K) with window expanded out from +4000 to -4000 ppm. This spectrum is the sum of 40 spectra, each with sw = 200 ppm. Artifacts from the instrument can be observed at +400 and -400 ppm (marked with a red asterisk). No hydride ^{1}H NMR signal could be located.

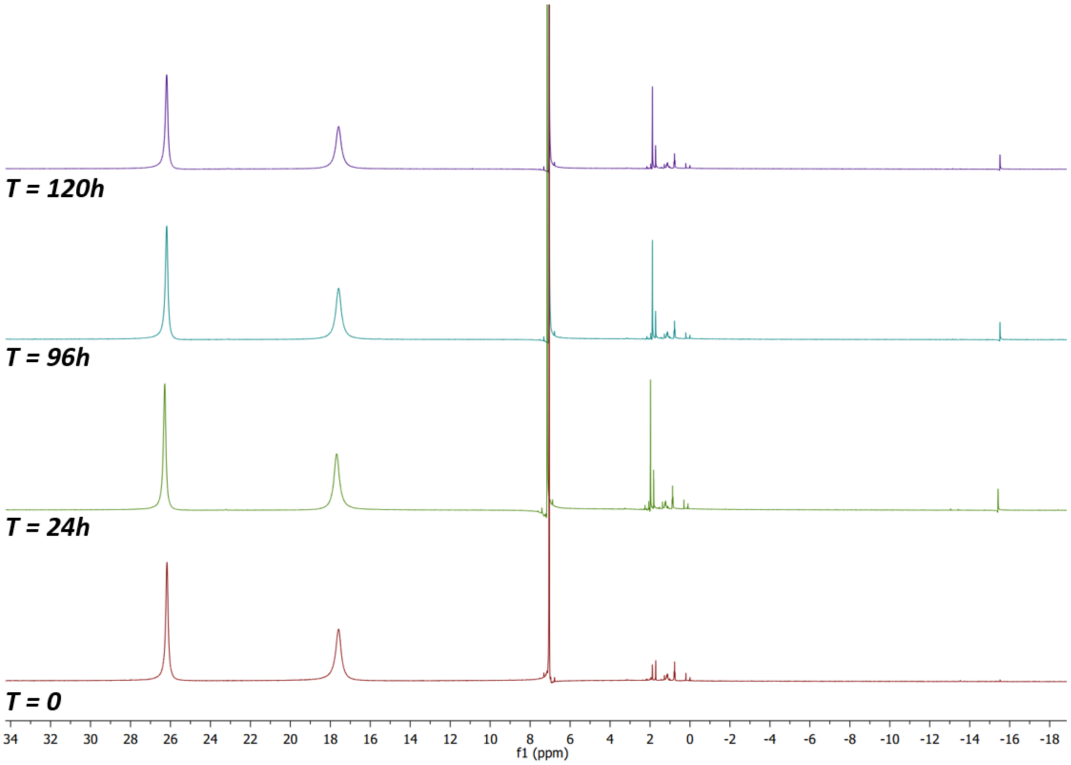

Figure S 4 : ^{1}H NMR monitoring of the stability of complex 1 over time in C_6D_6 solution while heating to 70°C. Spectra collected at 298K, 300 MHz.

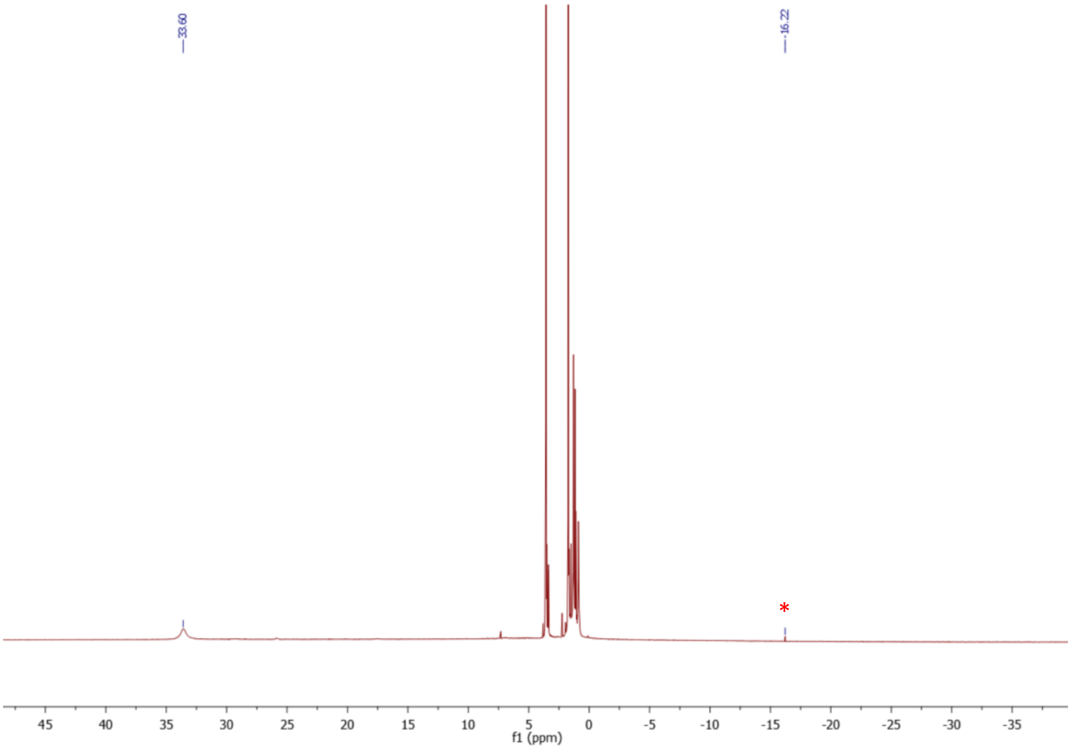

Figure S 5 : ^{1}H NMR spectrum (THF-*d*$_8$, 298 K, 300 MHz) of the crude reaction mixture of complex 1 + 2 eq. KC_8, showing the full consumption of complex 1 and the formation of complex 2 (Cp* broad resonance at +33.6 ppm). Some Cp*IrH_4 can be detected at -16 ppm and is marked with a red asterisk.

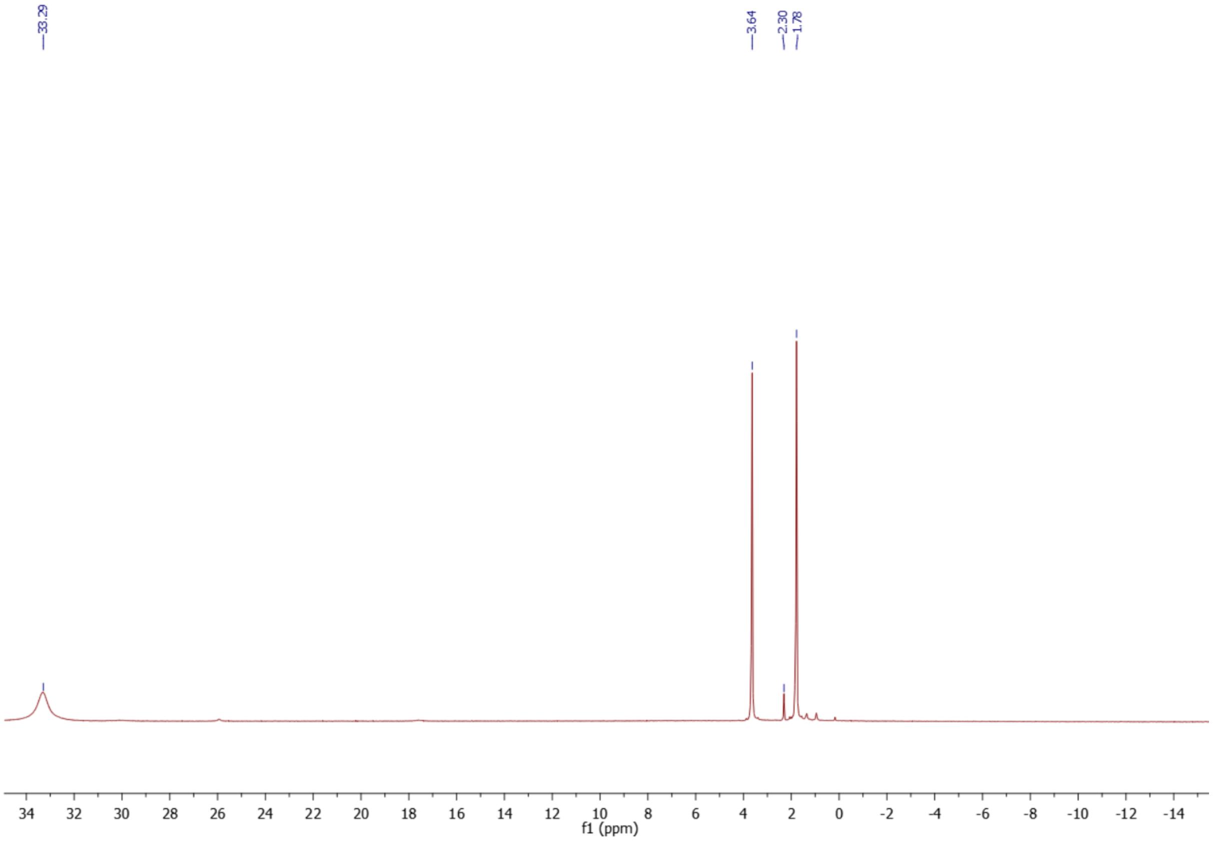

Figure S 6 : ^{1}H NMR spectrum of complex 2 recorded in THF-*d*$_8$ solution (300 MHz, 298 K).

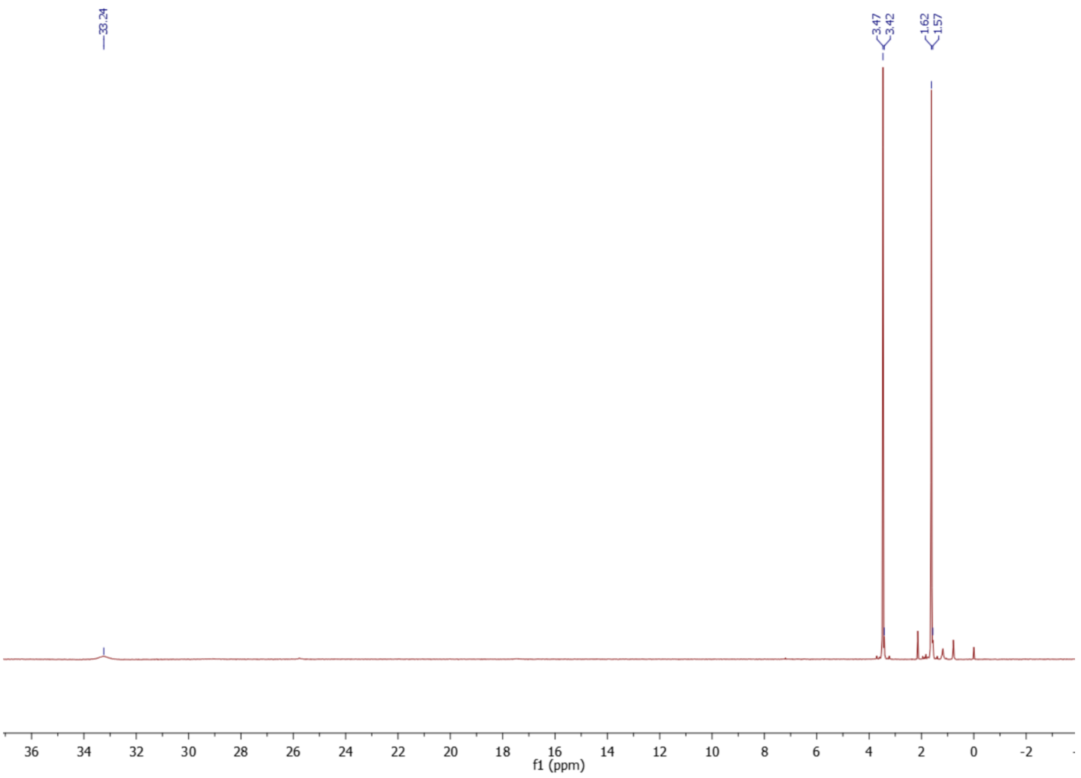

Figure S 7: ^{1}H NMR spectrum (300 MHz, 298 K) of complex 2 in THF-d_8 solution (1.2 mM) with a capillary THF-d_8 insert for Evans Method calculation of the magnetic moment. The THF signals have been shifted by 0.052 ppm (16.2 Hz) corresponding to a magnetic moment of 4.9 μ_B (4 unpaired electrons).

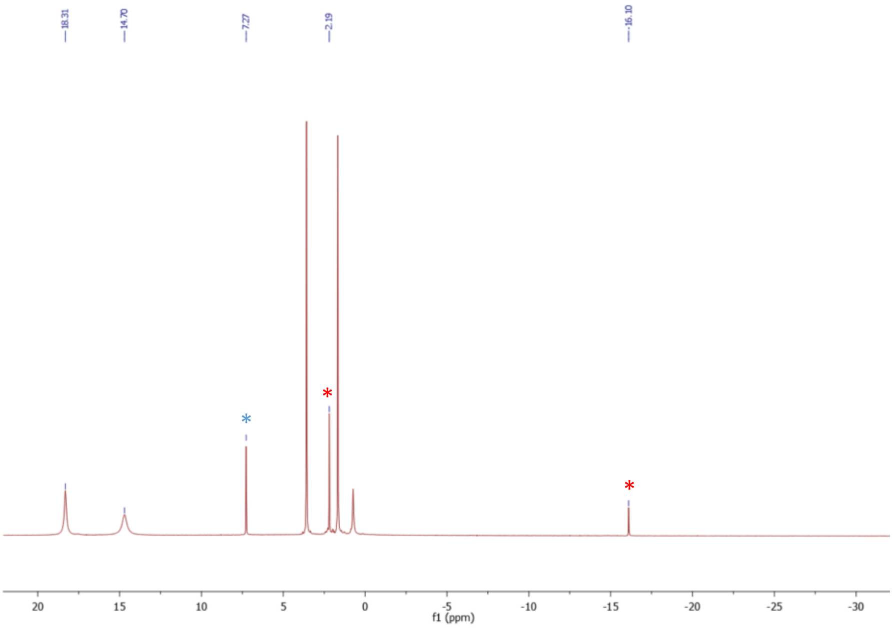

Figure S 8 : ^{1}H NMR spectrum of the crude reaction of 1 with 2 eq. $AgPF_6$ in THF-d_8 (298 K, 300 MHz). Some Cp*IrH_4 is observed, marked with a red asterisk (*) and some C_6D_6 marked with a blue asterisk.

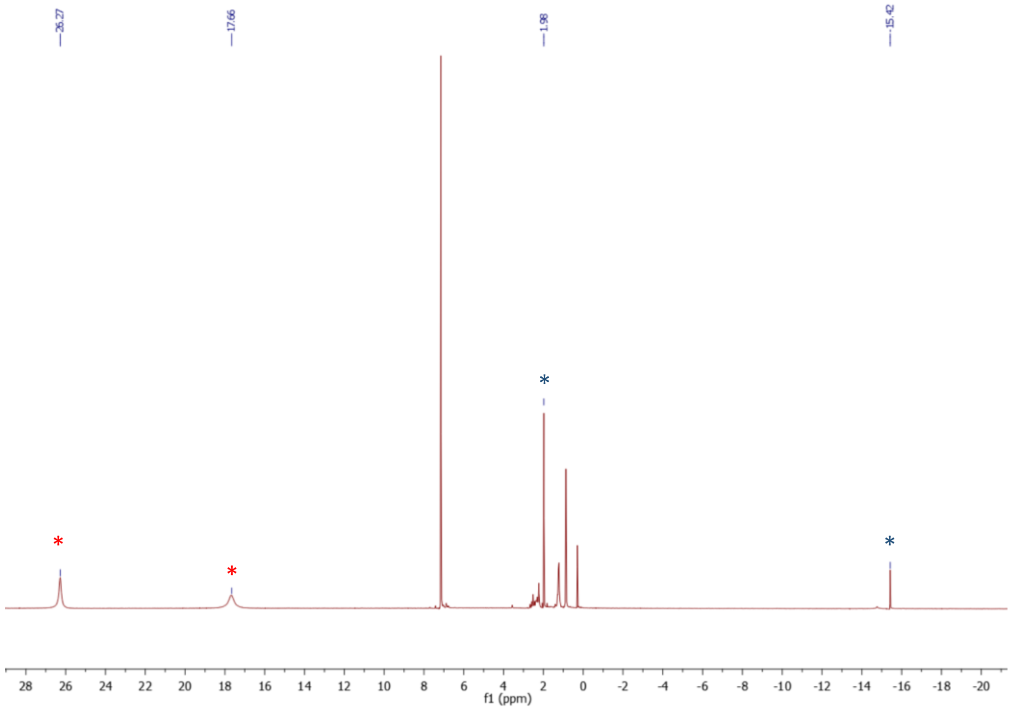

Figure S9 : ^{1}H NMR spectrum of the crude reaction mixture from the attempted oxidation of complex 2 with $AgPF_6$ in C_6D_6 (298 K, 300 MHz). The typical signals from complex 1 are observed (red asterisks). Some Cp*IrH_4 is also observed, as indicated by blue asterisks.

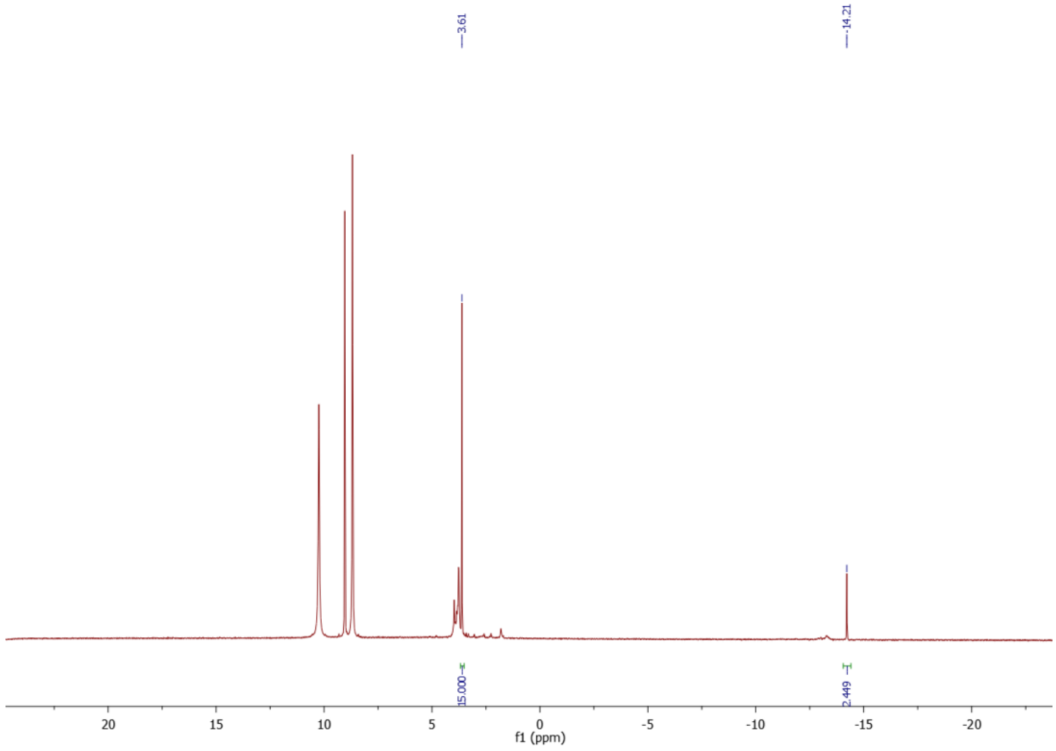

Figure S 10: ^{1}H NMR spectrum of the precipitate from the reaction of complex 2 with $AgPF_6$, dissolved in pyridine-*d*$_5$ (298 K, 300 MHz) confirming the production of the polymeric material [Cp*IrH_3Ag]$_n$. This spectrum matches literature expectation.[22]

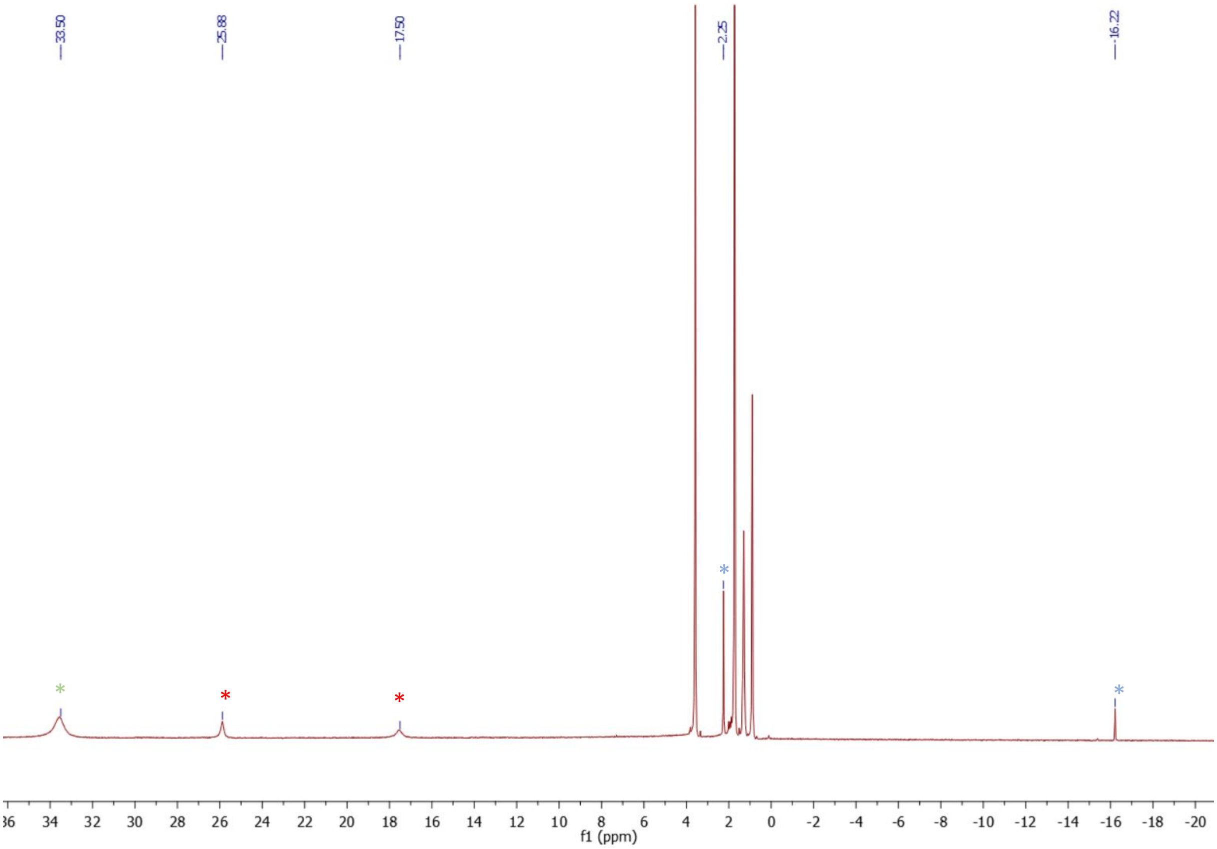

Figure S 11: A representative ^{1}H NMR spectrum of attempted oxidations of compound 2, here with XeF_2 in THF-d_8 (298 K, 300 MHz). Compound 1 is marked with a red asterisk, compound 2 marked with a green asterisk and Cp*IrH_4 is marked with a blue asterisk.

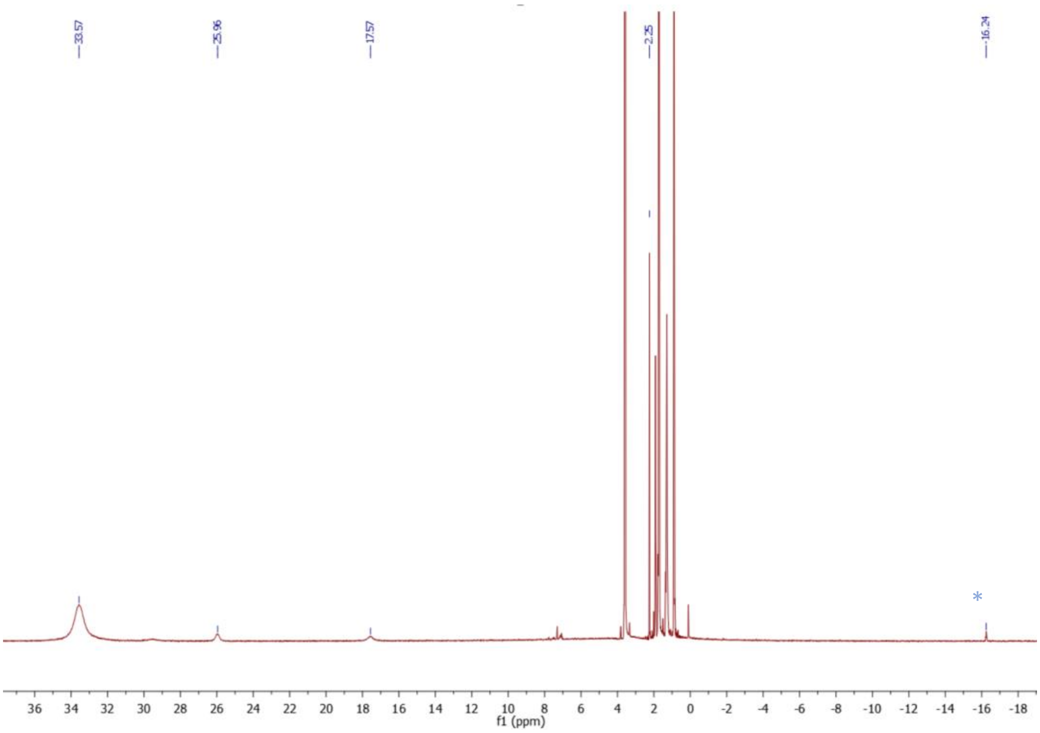

Figure S 12: Compound 2 is not stable in the presence of acetonitrile. Here, 2 was taken up in THF-d_8 (^{1}H NMR spectrum recorded at 298 K, 300 MHz) and a few drops of CD_3CN added and left at room temperature over 3 days. Compound 1 is marked with a red asterisk, Cp*IrH_4 is marked with a blue asterisk.

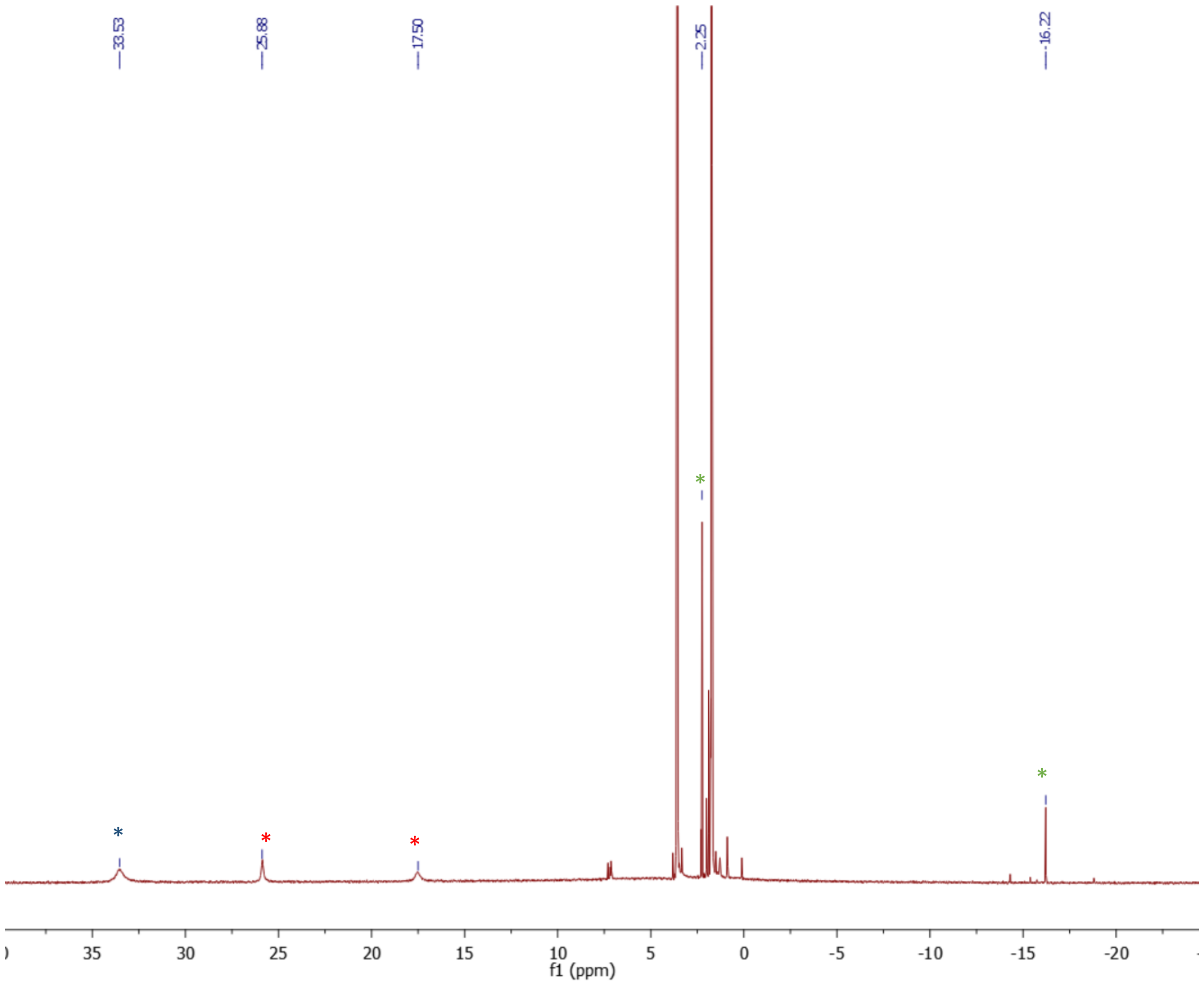

Figure S 13: ^{1}H NMR spectrum (THF-d_8, 300 MHz, 298 K) of the crude reaction mixture between $FeCl_3$ and three equivalents of K[Cp*IrH_3]. Complex 1 can be observed (as represented by red asterisks) along with complex 2 (blue asterisk) and Cp*IrH_4 (green asterisk).

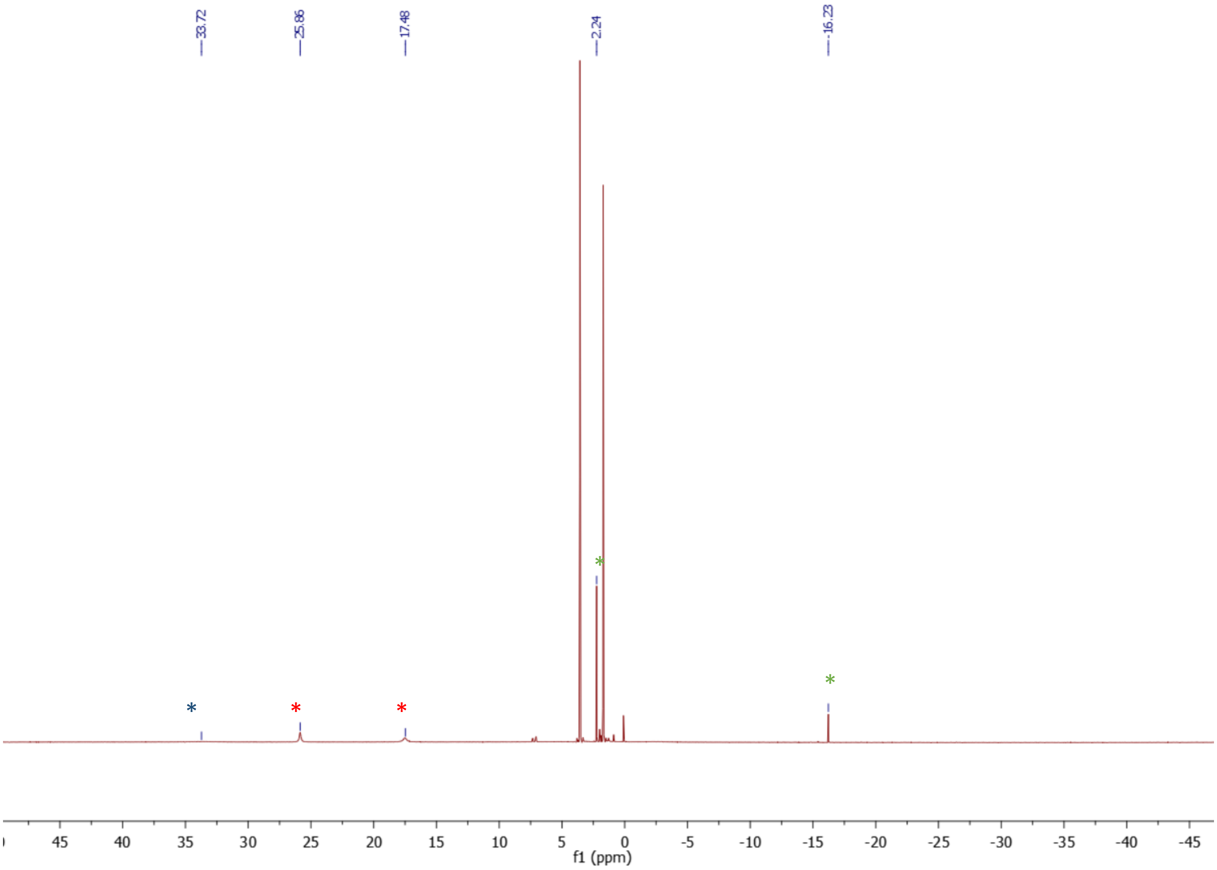

Figure S 14 : ^{1}H NMR spectrum (THF-d_8, 300 MHz, 298 K) of the crude reaction mixture between Fe(HMDS)$_3$ and three equivalents of Cp*IrH$_4$. Complex 1 can be observed (as represented by red asterisks) along with complex 2 (blue asterisk) and Cp*IrH$_4$ (green asterisk).

Diffuse Reflectance Infrared Transmission (DRIFT) Spectra

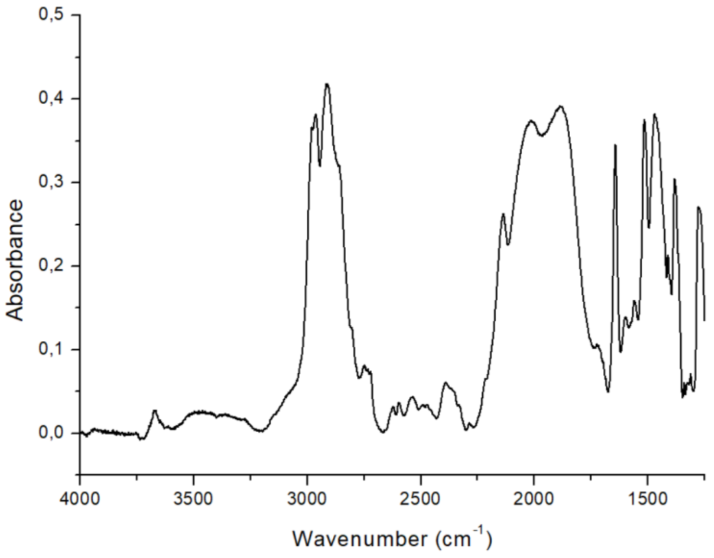

Figure S 15: DRIFT spectrum of compound 1 collected as a pure crystalline powder

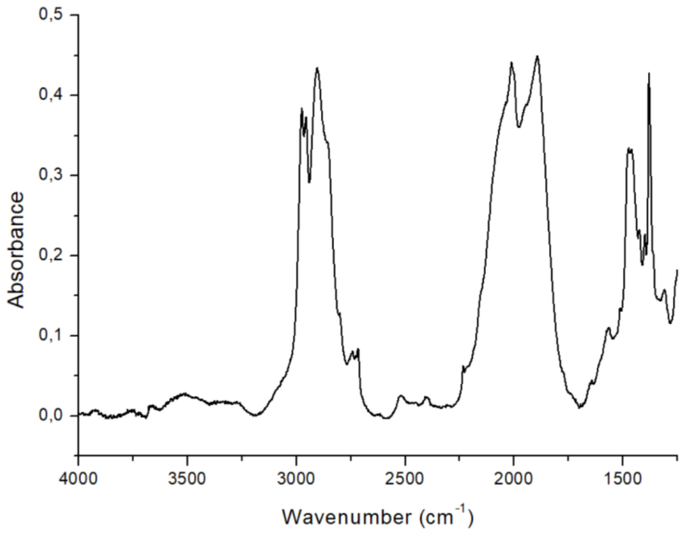

Figure S 16: DRIFT spectrum of compound 2 collected as a pure crystalline powder

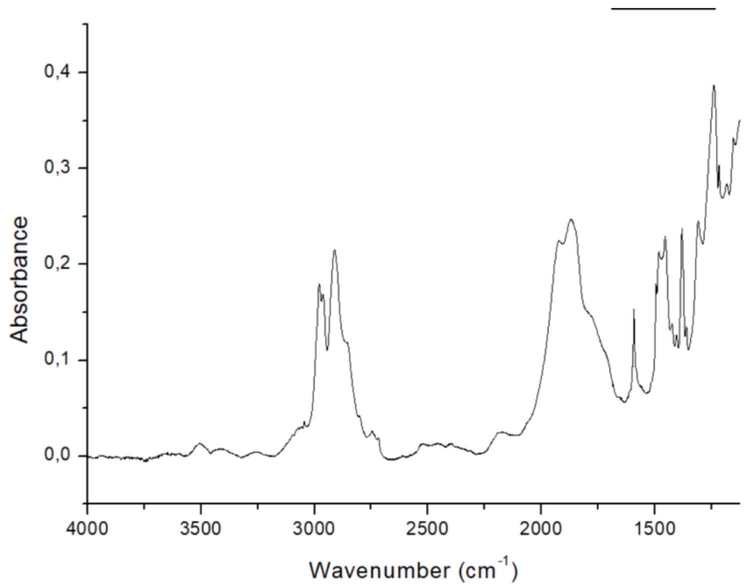

Figure S 17 : DRIFT spectrum of the crude product from attempted oxidation of 1 with two equivalents of $AgPF_6$.

Supplementary X-Ray Crystal Structures

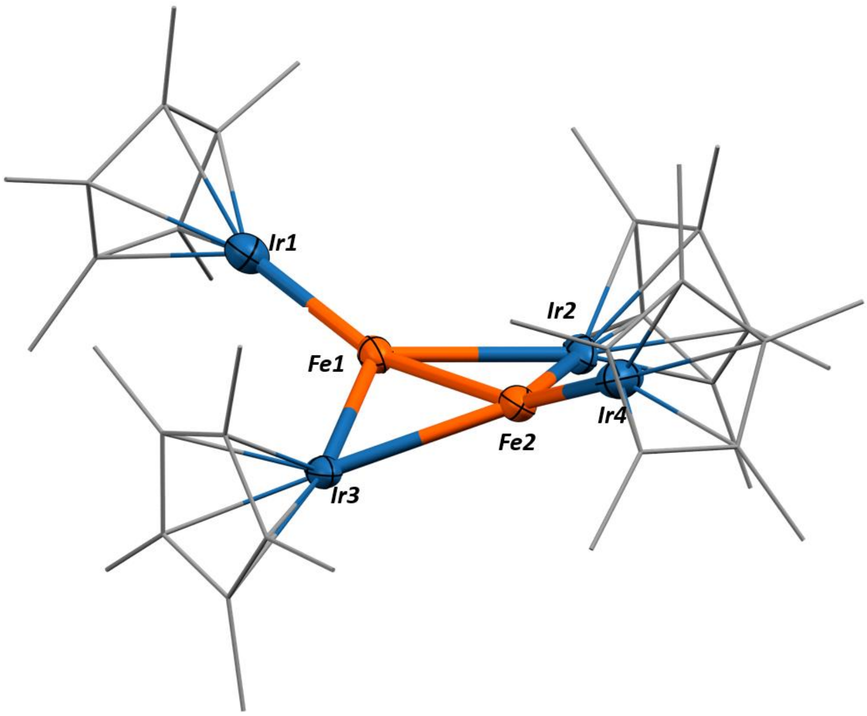

Figure S 18 : Another view of the X-ray diffraction structure of compound 1, showing the saddle shaped Fe_2Ir_4 core. Hydrogen atoms removed and Cp* ligands shown in wireframe for clarity.

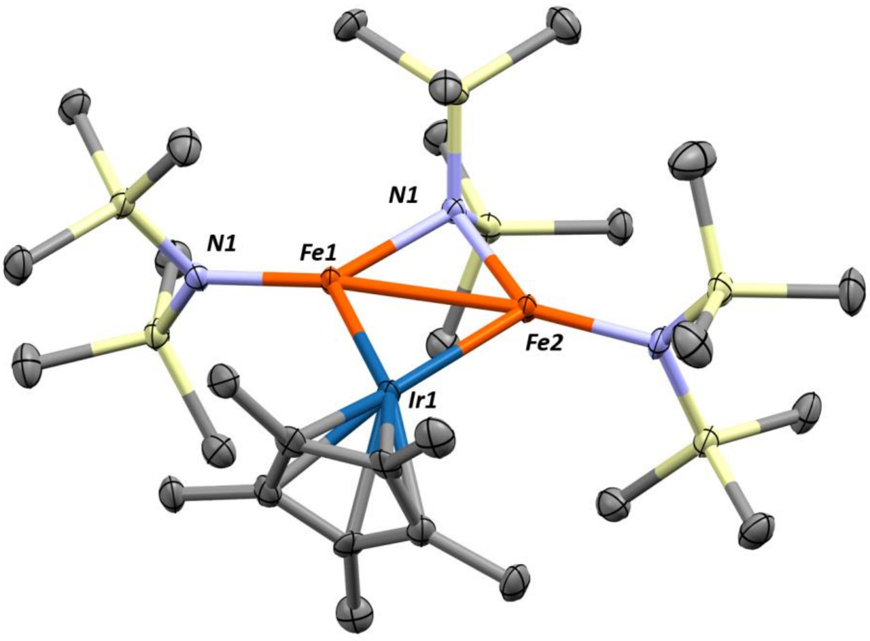

Figure S 19 : A substoichiometric addition of Cp^*IrH_4 to $Fe(HMDS)_2(THF)$ yields different products. A crystal was collected from one such reaction (0.5 Ir to 1 Fe equivalents, crystals grown from a saturated pentane solution) which shows an Fe_2Ir stoichiometry. Relevant structural parameters are as follows: Ir1 – Fe1 = 2.653(1), Ir1 – Fe2 = 2.640(1), Fe1 – Fe2 = 2.863(1), Fe1 – N1 = 1.940(6), Fe1 – N2 = 2.098(6), <$Cp^*_{Centroid}$ – Ir1 – Fe1Fe2$_{centroid}$ = 164.07, <Fe1 – Ir1 – Fe2 = 65.49(3), <Ir1 – Fe1 – N1 = 122.6(2), <Ir1 – Fe1 – Fe2 – N2 = 167.5(2)

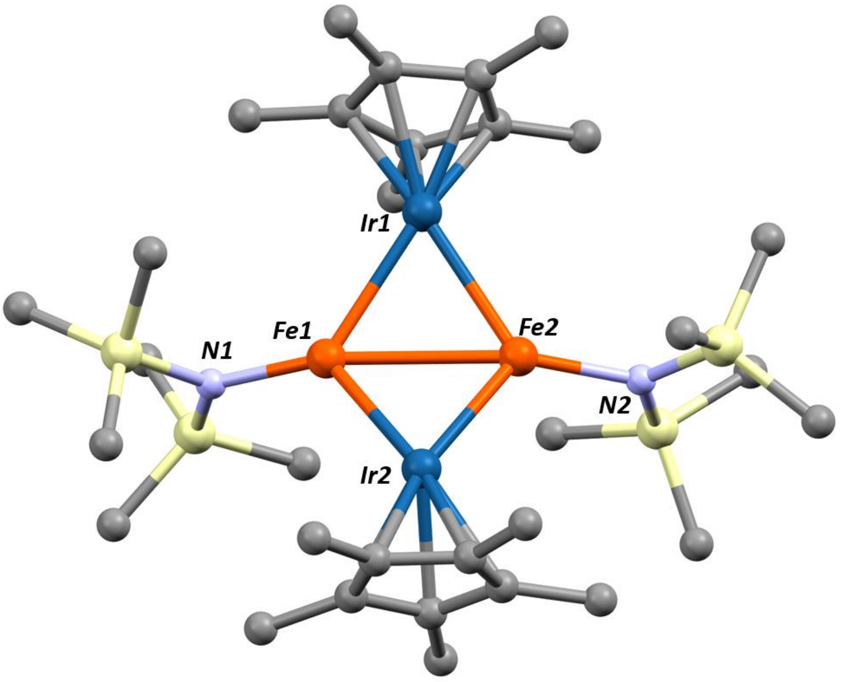

Figure S 20 : Addition of 1 eq. Cp*IrH_4 to 1 eq. $Fe(HMDS)_2(THF)$ yields different a Fe_2Ir_2 complex. A connectivity map could be obtained by XRD and is displayed as a ball and stick model for clarity. We see both iridiums bridging the iron centers, and silylamide ligands in the terminal positions. Unfortunately, the data from this XRD collection is not of publishable quality and so structural parameters cannot be accurately given here.

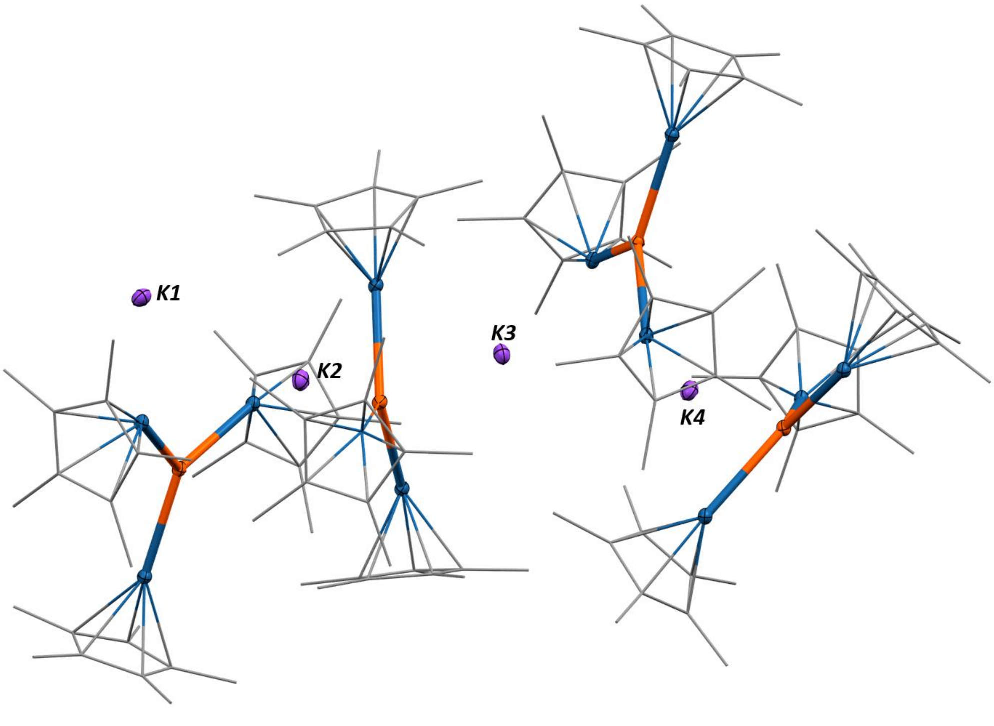

Figure S 21 : X-ray diffraction structure of complex 2, adopting a 1-D infinite chain structure in the solid state where potassium cations are connected by $FeIr_3$ fragments.

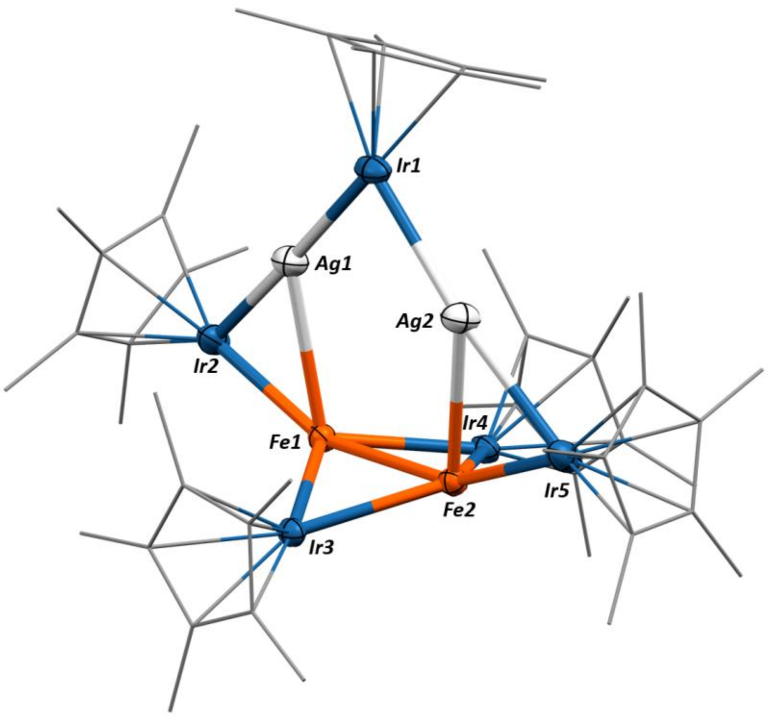

Figure S 22 : The structure of a cationic silver adduct of complex 1 found after attempted oxidation of the complex with $AgPF_6$. Crystals grown from a fluorobenzene:pentane vapour diffusion. A solvent mask was calculated and 1057 electrons were found in a volume of 3188 A^3 in 1 void *per* unit cell. This is compatible with the presence of two PF_6^- and two fluorobenzene molecules *per* Formula Unit, which account for 952 electrons *per* unit cell.

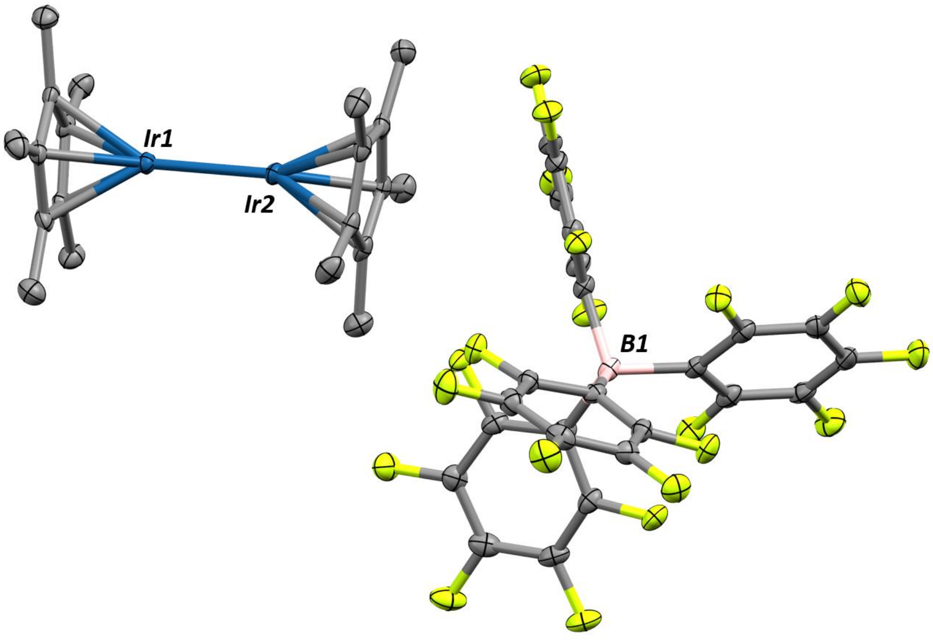

Figure S 23 : X-ray diffraction structure of the co-product from the oxidation of 2 with Magic Blue ([ArN]BArF), showing the Ir(III) dimer $[Cp^*Ir(\mu\text{-}H)_3IrCp^*]^+$, with a BArF counteranion. Relevant structural parameters are as follows: Ir1 – Ir2 = 2.455(6), <$Cp^*_{Centroid}$ – Ir1 – Ir2 = 177.3(1)°. Similar structures have been reported before with matching bond lengths confirming three hydrides present around the Ir-Ir bond thus confirming the Ir(III) oxidation state.[23,24]

X-Ray Crystallography Tables

**Compound**	**1**	**2**	**$(Cp^*IrH_3)Fe_2(HMDS)_3$**
Formula	$C_{40}H_{60}Fe_2Ir_4$	$C_{30}H_{45}FeIr_3K$	$C_{28}H_{69}Fe_2IrN_3Si_6$
$D_{calc.}$/ g cm^{-3}	2.108	2.043	1.434
μ/mm^{-1}	12.493	11.904	3.975
Formula Weight	1421.38	1077.21	920.30
Colour	dark yellow	black	dark orange
Shape	plate-shaped	needle-shaped	needle-shaped
Size/mm^3	0.33×0.26×0.02	0.22×0.02×0.01	0.26×0.12×0.10
T/K	100.0(2)	100.00(10)	100.00(19)
Crystal System	orthorhombic	tetragonal	monoclinic
Space Group	*Pbca*	$I4_1/acd$	$P2_1/c$
a/Å	15.8122(2)	29.4947(5)	16.0036(3)
b/Å	21.5429(3)	29.4947(5)	14.8911(2)
c/Å	26.2958(5)	16.1014(4)	17.8866(3)
α/°	90	90	90
β/°	90	90	90.2800(10)
γ/°	90	90	90
V/Å^3	8957.5(2)	14007.2(6)	4262.53(12)
Z	8	16	4
Z'	1	0.5	1
Wavelength/Å	0.71073	0.71073	0.71073
Radiation type	Mo K_α	Mo K_α	Mo K_α
Θ_{min}/°	2.415	2.712	2.545
Θ_{max}/°	30.484	30.594	30.391
Measured Refl's.	104247	39144	98492
Indep't Refl's	11979	4849	11432
Refl's I≥2 σ(I)	8731	3665	9514
R_{int}	0.0783	0.0987	0.0826
Parameters	531	215	385
Restraints	780	236	135
Largest Peak	5.910	2.090	3.145
Deepest Hole	-2.775	-1.306	-4.441
GooF	1.087	1.170	1.181
wR_2 (all data)	0.1647	0.1144	0.1745
wR_2	0.1447	0.1067	0.1565
R_1 (all data)	0.1043	0.1047	0.0677
R_1	0.0697	0.0705	0.0536

**Compound**	**1 + 2 eq. $AgPF_6$**	**[Cp*Ir(μ-H_3)IrCp*]BArF**
Formula	$C_{62}H_{70}Ag_2F_{14}Fe_2Ir_5P_2$	$C_{44}H_{30}BF_{20}Ir_2$
$D_{calc.}$/ g cm^{-3}	2.003	2.064
μ/mm^{-1}	9.142	12.931
Formula Weight	2431.56	1333.89
Colour	brown	yellow
Shape	needle-shaped	needle-shaped
Size/mm^3	0.14×0.05×0.02	0.19×0.06×0.02
T/K	100.00(10)	100.0(2)
Crystal System	monoclinic	monoclinic
Space Group	$C2/m$	$P2_1/n$
a/Å	30.8995(6)	10.76590(10)
b/Å	18.7898(2)	16.58320(10)
c/Å	17.3078(3)	24.42680(10)
α/°	90	90
β/°	126.636(3)	100.1450(10)
γ/°	90	90
V/Å^3	8063.6(3)	4292.81(5)
Z	4	4
Z'	0.5	1
Wavelength/Å	0.71073	1.54184
Radiation type	Mo K$_\alpha$	Cu K$_\alpha$
Θ_{min}/°	3.202	3.237
Θ_{max}/°	30.528	78.947
Measured Refl's.	93165	83185
Indep't Refl's	11258	8955
Refl's I≥2 σ(I)	9350	8674
R_{int}	0.0378	0.0334
Parameters	377	615
Restraints	607	0
Largest Peak	4.056	0.788
Deepest Hole	-0.856	-0.888
GooF	1.100	1.113
wR_2 (all data)	0.0783	0.0553
wR_2	0.0762	0.0549
R_1 (all data)	0.0366	0.0205
R_1	0.0286	0.0198

Supplementary ESI-MS data

CENTRE COMMUN DE SPECTROMETRIE DE MASSE

Analysis Info

Analysis Name	Impact2_250829_01_ZD551.d		
Method	Tune_pos_Standard.m	Acquisition Date	8/29/2025 10:17:11 AM
Comment		Instrument / Ser#	impact II 1825265.10081

Acquisition Parameter

Source Type	ESI	Ion Polarity	Positive	Set Nebulizer	0.3 Bar
Focus	Active	Set Capillary	4500 V	Set Dry Heater	200 °C
Scan Begin	50 m/z	Set End Plate Offset	-500 V	Set Dry Gas	4.0 l/min
Scan End	1500 m/z	Set Collision Cell RF	750.0 Vpp	Set Divert Valve	Source

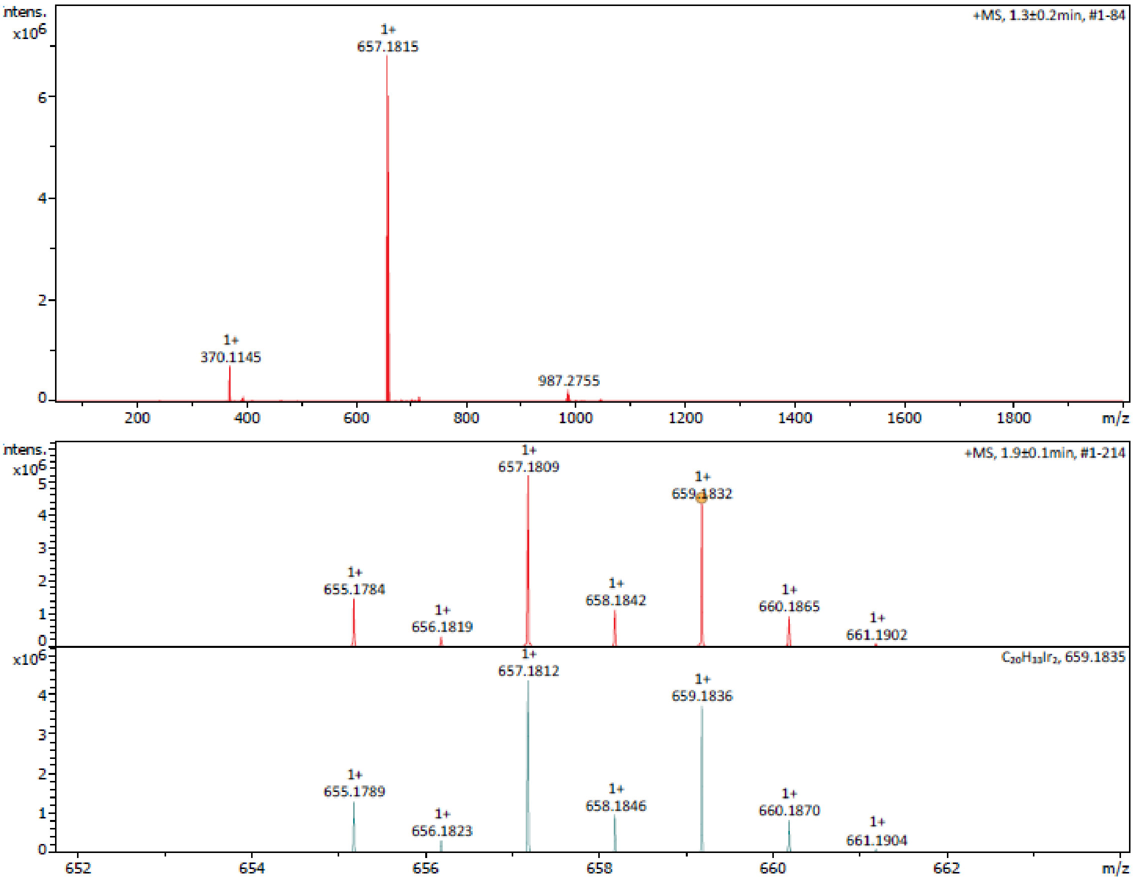

Meas. m/z	Ion Formula	m/z	Sum Formula	err [ppm]	mSigma	Adduct	z
659.1832	C20H33Ir2	659.1835	C20H33Ir2	0.6	9.9	M	1+
659.1832	C20H33Ir2	659.1835	C20H32Ir2	0.6	9.9	M+H	1+
659.1832	C18H34Ir2Na	659.1811	C18H34Ir2	-3.1	12.4	M+Na	1+

Figure S 24 : ESI-MS spectrum recorded for compound 1, showing a signal at m/z = 657.18 corresponding to the [Cp*IrH$_3$IrCp*]$^+$ cation, which we assume is a decomposition product from the ionization

CENTRE COMMUN DE SPECTROMETRIE DE MASSE

Analysis Info

Analysis Name	Impact2_250829_02_ZD552.d		
Method	Tune_pos_Standard.m	Acquisition Date	8/29/2025 10:41:39 AM
Comment		Instrument / Ser#	impact II 1825265.1 0081

Acquisition Parameter

Source Type	ESI	Ion Polarity	Positive	Set Nebulizer	0.3 Bar
Focus	Active	Set Capillary	4500 V	Set Dry Heater	200 °C
Scan Begin	50 m/z	Set End Plate Offset	-500 V	Set Dry Gas	4.0 l/min
Scan End	2000 m/z	Set Collision Cell RF	750.0 Vpp	Set Divert Valve	Source

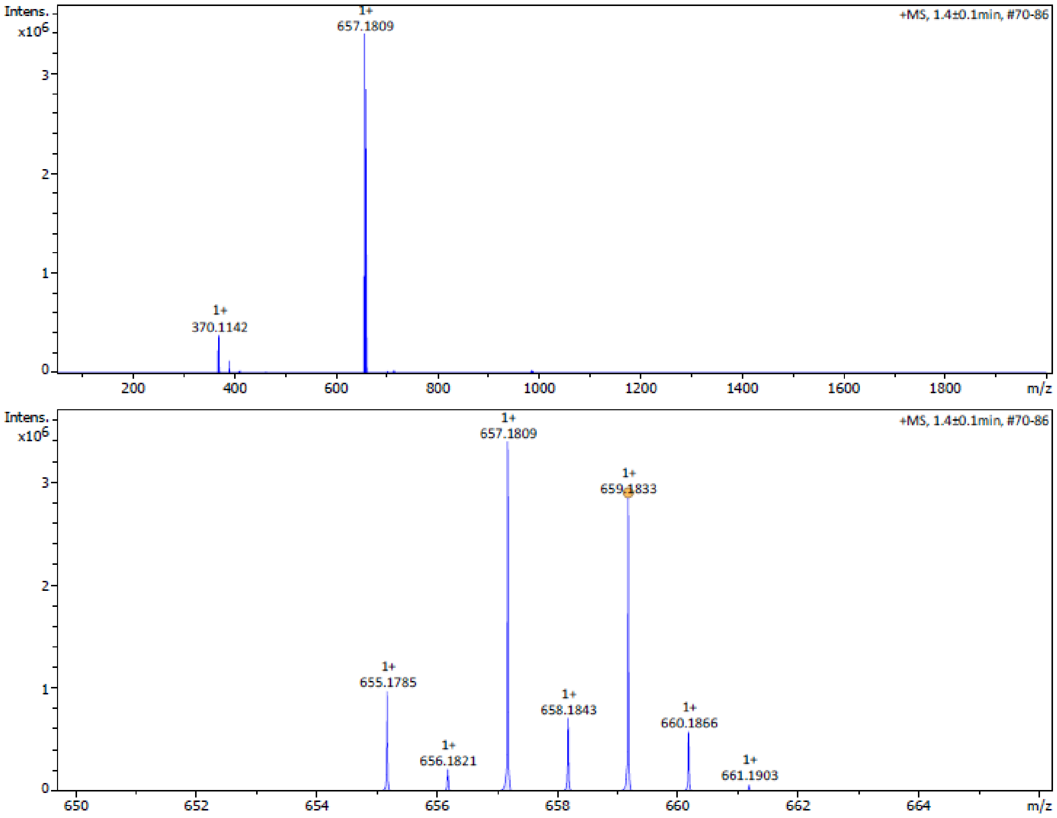

Meas. m/z	Ion Formula	m/z	Sum Formula	err [ppm]	mSigma	Adduct	z
659.1833	C20H33Ir2	659.1835	C20H33Ir2	0.4	11.1	M	1+
659.1833	C20H33Ir2	659.1835	C20H32Ir2	0.4	11.1	M+H	1+
659.1833	C18H34Ir2Na	659.1811	C18H34Ir2	-3.2	8.7	M+Na	1+

Figure S 25 : ESI-MS spectrum recorded for compound 2, showing a signal at m/z = 657.18 corresponding to the [Cp*IrH$_3$IrCp*]$^+$ cation, which we assume is a decomposition product from the ionization

References

(1) Bau, R.; Ho, D. M.; Gibbins, S. G. The Binary Metal Hydrido Anion Hexahydroferrate(4-). X-Ray Structural Characterization. *J. Am. Chem. Soc.* **1981**, *103* (16), 4960–4962. https://doi.org/10.1021/ja00406a058.

(2) Araake, R.; Sakadani, K.; Tada, M.; Sakai, Y.; Ohki, Y. [Fe4] and [Fe6] Hydride Clusters Supported by Phosphines: Synthesis, Characterization, and Application in N2 Reduction. *J. Am. Chem. Soc.* **2017**, *139* (15), 5596–5606. https://doi.org/10.1021/jacs.7b01965.

(3) Reiners, M.; Maekawa, M.; Baabe, D.; Zaretzke, M.-K.; Schweyen, P.; Daniliuc, C. G.; Freytag, M.; Raeder, J.; Hohenberger, J.; Sutter, J.; Meyer, K.; Walter, M. D. Monomeric Fe(III) Half-Sandwich Complexes [Cp′FeX2] – Synthesis, Properties and Electronic Structure. *Dalton Trans.* **2018**, *47* (31), 10517–10526. https://doi.org/10.1039/C8DT01570H.

(4) Tseng, C.-C.; Ding, Y.-W.; Chen, Z.-Y.; Lan, H.-Y.; Li, H.-J.; Cheng, Y.-S.; Kuo, T.-S.; Chen, P.-L.; Wu, W.-C.; Shi, F.-K.; Yang, T.; Liu, H.-J. A Bis-Cyclopentadienyl Ligand-Supported Di-Iron Trihydride Motif as a Synthon for Access to Heterobimetallic Trinuclear Complexes. *Inorg. Chem.* **2024**, *63* (24), 11361–11368. https://doi.org/10.1021/acs.inorgchem.4c01420.

(5) Guilera, G.; McGrady, G. S.; Steed, J. W.; Kaltsoyannis, N. Expanding Metallaborane Chemistry: An Octahedral BH6 Moiety Supported through M–H–B Bridges. *New J. Chem.* **2004**, *28* (4), 444–446. https://doi.org/10.1039/B401442C.

(6) Gorgas, N.; Stöger, B.; Veiros, L. F.; Kirchner, K. Access to FeII Bis(σ-B−H) Aminoborane Complexes through Protonation of a Borohydride Complex and Dehydrogenation of Amine-Boranes. *Angewandte Chemie International Edition* **2019**, *58* (39), 13874–13879. https://doi.org/10.1002/anie.201906971.

(7) Ishihara, K.; Araki, Y.; Tada, M.; Takayama, T.; Sakai, Y.; Sameera, W. M. C.; Ohki, Y. Synthesis of Dinuclear Mo−Fe Hydride Complexes and Catalytic Silylation of N2. *Chemistry – A European Journal* **2020**, *26* (43), 9537–9546. https://doi.org/10.1002/chem.202000104.

(8) Oishi, M.; Endo, T.; Oshima, M.; Suzuki, H. Aluminum-Stabilized Low-Spin Iron(II) Hydrido Complexes of 1,4,7-Trimethyl-1,4,7-Triazacyclononane. *Inorg. Chem.* **2014**, *53* (10), 5100–5108. https://doi.org/10.1021/ic500195q.

(9) Plois, M.; Hujo, W.; Grimme, S.; Schwickert, C.; Bill, E.; de Bruin, B.; Pöttgen, R.; Wolf, R. Open-Shell First-Row Transition-Metal Polyhydride Complexes Based on the Fac-[RuH3(PR3)3]− Building Block. *Angewandte Chemie International Edition* **2013**, *52* (4), 1314–1318. https://doi.org/10.1002/anie.201205209.

(10) Perez-Jimenez, M.; Crimmin, M. R. Photochemical H2 Activation by an Zn–Fe Heterometallic: A Mechanistic Investigation. *Chem. Sci.* **2024**, *15* (4), 1424–1430. https://doi.org/10.1039/D3SC05966A.

(11) Ott, J. C.; Blasius, C. K.; Wadepohl, H.; Gade, Lutz. H. Synthesis, Characterization, and Reactivity of a High-Spin Iron(II) Hydrido Complex Supported by a PNP Pincer Ligand and Its Application as a Homogenous Catalyst for the Hydrogenation of Alkenes. *Inorg. Chem.* **2018**, *57* (6), 3183–3191. https://doi.org/10.1021/acs.inorgchem.7b03227.

(12) Gilbert, T. M.; Hollander, F. J.; Bergman, R. G. (Pentamethylcyclopentadienyl)Iridium Polyhydride Complexes: Synthesis of Intermediates in the Mechanism of Formation of (Pentamethylcyclopentadienyl)Iridium Tetrahydride and the Preparation of Several Iridium(V) Compounds. *J. Am. Chem. Soc.* **1985**, *107* (12), 3508–3516. https://doi.org/10.1021/ja00298a018.

(13) Broere, D. L. J.; Čorić, I.; Brosnahan, A.; Holland, P. L. Quantitation of the THF Content in Fe[N(SiMe3)2]2·xTHF. *Inorg. Chem.* **2017**, *56* (6), 3140–3143. https://doi.org/10.1021/acs.inorgchem.7b00056.

(14) Ye, C. Z.; Rosal, I. D.; Boreen, M. A.; Ouellette, E. T.; Russo, D. R.; Maron, L.; Arnold, J.; Camp, C. A Versatile Strategy for the Formation of Hydride-Bridged Actinide–Iridium Multimetallics. *Chem. Sci.* **2023**, *14* (4), 861–868. https://doi.org/10.1039/D2SC04903A.

(15) Bradley, D. C.; Copperthwaite, R. G.; Extine, M. W.; Reichert, W. W.; Chisholm, M. H. Transition Metal Complexes of Bis(Trimethyl-Silyl)Amine (1,1,1,3,3,3-Hexamethyldisilazane). In *Inorganic Syntheses*; John Wiley & Sons, Ltd, 1978; pp 112–120. https://doi.org/10.1002/9780470132494.ch18.

(16) O'Connor, A. R.; Nataro, C.; Golen, J. A.; Rheingold, A. L. Synthesis and Reactivity of [N(C6H4Br)3][B(C6F5)4]: The X-Ray Crystal Structure of [Fe(C5H5)2][B(C6F5)4]. *Journal of Organometallic Chemistry* **2004**, *689* (14), 2411–2414. https://doi.org/10.1016/j.jorganchem.2004.04.034.

(17) *CrysAlisPro Software System*; Rigaku Oxford Diffraction.

(18) Clark, R. C.; Reid, J. S. The Analytical Calculation of Absorption in Multifaceted Crystals. *Acta Cryst A* **1995**, *51* (6), 887–897. https://doi.org/10.1107/S0108767395007367.

(19) Sheldrick, G. M. SHELXT – Integrated Space-Group and Crystal-Structure Determination. *Acta Cryst A* **2015**, *71* (1), 3–8. https://doi.org/10.1107/S2053273314026370.

(20) Dolomanov, O. V.; Bourhis, L. J.; Gildea, R. J.; Howard, J. a. K.; Puschmann, H. OLEX2: A Complete Structure Solution, Refinement and Analysis Program. *J Appl Cryst* **2009**, *42* (2), 339–341. https://doi.org/10.1107/S0021889808042726.

(21) Bain, G. A.; Berry, J. F. Diamagnetic Corrections and Pascal's Constants. *J. Chem. Educ.* **2008**, *85* (4), 532. https://doi.org/10.1021/ed085p532.

(22) Escomel, L.; Soulé, N.; Robin, E.; Del Rosal, I.; Maron, L.; Jeanneau, E.; Thieuleux, C.; Camp, C. Rational Preparation of Well-Defined Multinuclear Iridium–Aluminum Polyhydride Clusters and Comparative Reactivity. *Inorg. Chem.* **2022**, *61* (15), 5715–5730. https://doi.org/10.1021/acs.inorgchem.1c03120.

(23) White, C.; Oliver, A. J.; Maitlis, P. M. Pentamethylcyclopentadienyl-Rhodium and -Iridium Complexes. Part VII. Mono-, Di-, and Tri-μ-Hydrido-Complexes. *J. Chem. Soc., Dalton Trans.* **1973**, No. 18, 1901–1907. https://doi.org/10.1039/DT9730001901.

(24) Gilbert, T. M.; Bergman, R. G. Synthesis of Trimethylphosphine-Substituted (Pentamethylcyclopentadienyl)Iridium Hydride Complexes; Protonation and Deprotonation of (Pentamethylcyclopentadienyl)(Trimethylphosphine)Iridium Dihydride. *J. Am. Chem. Soc.* **1985**, *107* (12), 3502–3507. https://doi.org/10.1021/ja00298a017.
